# StereoPylot: An Open-Source Raspberry Pi-Based Stereotaxic Apparatus Controller with 3D Printed Components for Fully Motorized Control, Digital Display, and Customizable Features

**DOI:** 10.1523/ENEURO.0460-25.2026

**Published:** 2026-07-07

**Authors:** Kirk Mulatz, Aiden E. Glass, McKenna A. Bolger, Caleb L. G. White, John G. Howland, Justin J. Botterill

**Affiliations:** Department of Anatomy, Physiology, and Pharmacology, University of Saskatchewan, Saskatoon, Saskatchewan S7N 5E5, Canada

**Keywords:** 3D printing, adeno-associated virus, anatomy, brain, neuroscience, Raspberry Pi, stereotaxic apparatus

## Abstract

Stereotaxic surgery is a powerful technique used to deliver drugs, viral vectors, and/or probes into discrete regions of the brain. Over the past decade, the emphasis on performing complex surgeries for neural circuit manipulations or studying multiple brain regions in the same animal has increased. While analog stereotaxic instruments are generally suitable for these types of experiments, the surgeon must accurately read vernier scales for each brain region of interest, which is both time-consuming and prone to user error. Although vendors now offer digital stereotaxic instruments that overcome the limitations of analog instruments, digital units are cost-prohibitive and often come in limited configurations that lack clear paths to customize or upgrade as experimental needs change. To overcome these issues, we've created StereoPylot, an open-source Raspberry Pi-based stereotaxic controller with 3D-printed components. StereoPylot features motorized control of *X*-, *Y*-, and *Z*-axes, variable motor speed, programmable buttons, and a digital display for stereotaxic coordinates. We also created a custom graphical user interface which allows surgeons to move the manipulators to an area of interest by manually entering stereotaxic coordinates on screen or by loading a preset file that contains a list of user-defined stereotaxic coordinates. We demonstrate practical application of StereoPylot by quantifying viral expression in three male and three female Long–Evans rats and provide instrument accuracy and precision measurements. StereoPylot is therefore feature-rich and highly customizable while being relatively inexpensive to implement onto existing analog instruments commonly found in neuroscience laboratories.

## Significance Statement

We developed a fully motorized digital stereotaxic apparatus using a mixture of commercially available and 3D-printed components. StereoPylot is controlled by a Raspberry Pi device and features a 3D-printed controller box with customizable buttons and a large digital display. We designed StereoPylot so that it can be retrofitted onto a variety of 0.1 mm commercially available stereotaxic manipulators. StereoPylot provides repeatable accuracy and push button control of automated features designed to relieve the surgeon from having to perform repetitive motions and calculations which contribute to potential errors during stereotaxic surgery. The open-source nature of our design encourages users to further customize StereoPylot to accommodate their specific experimental needs.

## Introduction

Stereotaxic surgery is a fundamental technique in systems neuroscience that enables precise targeting of brain regions for experimental manipulations or measurements (Pritchett-Corning et al., 2011; Ferry et al., 2014). Stereotaxic surgical instruments utilize a three-dimensional (3D) Cartesian coordinate system (*X*-, *Y*-, *Z*-axes) that allow researchers to deliver drugs, viral vectors, and/or probes into the brains of small and large animals (Cecyn and Abrahao, 2023). Brain atlases for numerous species provide surgeons with 3D stereotaxic coordinates for regions of interest relative to key reference sites on the skull such as bregma or lambda (Paxinos and Watson, 1998; Cecyn and Abrahao, 2023). Modern stereotaxic instruments are offered by numerous vendors and feature common components such as a large base plate, a U-shaped frame, adjustable ear bars and an incisor bar to secure the head, a nose cone for delivering anesthetic drugs, and manipulator arms with built-in micrometers that hold and move tools in the medial–lateral (ML) and dorsal–ventral (DV) axes.

While the general components of stereotaxic instruments have remained consistent over time, advances in neuroscience tools and probes over the past decade have significantly increased the complexity of rodent stereotaxic surgeries (Buzsaki et al., 2015; Allen et al., 2020; Emiliani et al., 2022). Many laboratories now perform surgeries that involve multiple intracranial injections and/or probe implants (e.g., optical fibers, electrodes, tetrode arrays) across brain regions to manipulate and/or record neural activity (Simpson et al., 2024). However, as the complexity of surgical procedures increases, there is a greater risk for surgeon fatigue and error, which can affect surgical success (Allen et al., 2020). For example, standard analog stereotaxic instruments require the surgeon to accurately read vernier scales for each region of interest and perform stereotaxic coordinate calculations in real time, which is both time-consuming and increasingly prone to user errors. Several vendors now offer digital and motorized stereotaxic instruments that reduce the demands of the surgeon by providing large digital displays and ergonomic controls. However, digital and motorized stereotaxic instruments are cost-prohibitive and often come in limited configurations that lack flexible upgrade paths. The lack of highly customizable stereotaxic instruments is problematic to many researchers because experimental needs often change over time.

Consumer-level 3D printing technology is now widely available and allows researchers to rapidly create custom equipment or tools at a fraction of the cost and time of traditional manufacturing processes (Tek et al., 2008; Dhar et al., 2025). Several laboratories have taken advantage of 3D printing in the context of stereotaxic surgery. For example, 3D-printed models of rodent skulls and brains provide animal-free stereotaxic surgery training (Bainier et al., 2021). Several laboratories have also created custom 3D-printed stereotaxic components to support their research questions, such as adaptors to enable intracranial injections in neonatal or infant mice (Olivetti et al., 2020; Chervonski et al., 2021; Steffens et al., 2022), a self-targeting brain implant system for rats (Allen et al., 2020), components to make custom guide cannulas in-house (Tropea et al., 2022), and a 3D-printable frameless stereotaxic apparatus for pigs (Malbert, 2021). In-laboratory 3D printing has also been used to generate custom objects or mazes for rodent behavioral testing (Inayat et al., 2021; O’Leary et al., 2025; O’Neill et al., 2025; Porter et al., 2025), platforms for head-fixed in vivo recordings (Cristino et al., 2025), and chambers for ex vivo recordings (Withers et al., 2024).

**Figure 1.**
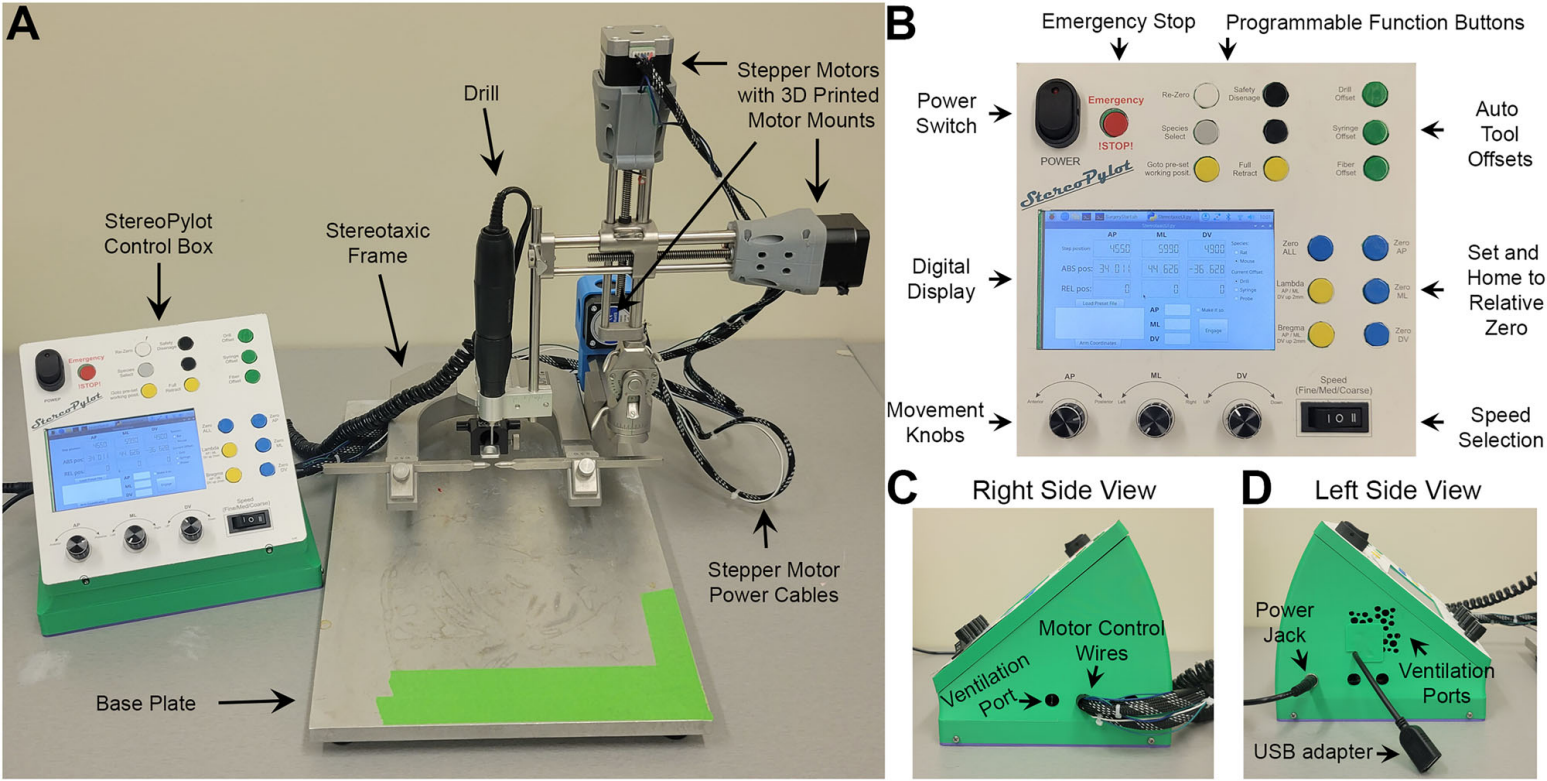
Overview of the StereoPylot stereotaxic instrument. ***A***, Representative photo showing an analog stereotaxic instrument that was modified to incorporate a StereoPylot controller box and motor mount hardware. ***B***, Control box face outlining the layout of the functions. StereoPylot features a large digital LCD display with a custom GUI, a power switch, an emergency stop button, programmable function buttons, programmable offset compensation buttons for tools or probes, zero and homing buttons, movement control via rotary knobs, and variable speed selection (coarse, medium, fine). ***C***, The right side view of the control box showing a ventilation port and access hole for the motor control wires. ***D***, The left side view of control box showing the ventilation ports, power jack connector, and USB adapter for transferring lists of preset coordinates.

**Figure 2.**
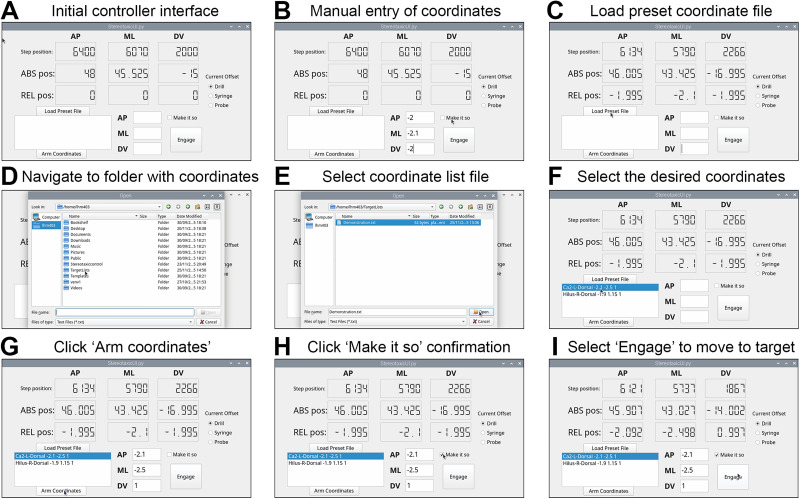
Overview of the StereoPylot GUI and custom features. ***A***, Initial controller interface with relative coordinates set to “0”. The assisted coordinate navigation feature of StereoPylot can be used by (***B***) entering the coordinates manually or (***C*–*G***) loading a precompiled list of coordinates from a text file downloaded to the Raspberry Pi. ***H***, Once the coordinates are loaded a safety check must be engaged by clicking the “Make it so” box. ***I***, Once the “Engage” button is pressed, the unit will navigate to the desired coordinates, relative to the set zero. Multiple coordinates can be navigated sequentially without having to re-zero.

In the present study, we used 3D printing technology, a low-cost and small format single-board computer (Raspberry Pi, #3B+), and commercially available electronic components to convert a standard analog stereotaxic instrument into a fully digital and motorized device called StereoPylot (Fig. 1; Movie 1). Our primary aim was to design a conversion kit that utilizes readily available and low-cost parts (total cost <$500 USD). We also added ease of use features to the controller box, such as programmable buttons and a feature-rich custom graphical user interface (GUI; Fig. 2). To ensure the maximum compatibility of our instrument, we created several 3D-printable motor mount files to accommodate a variety of stereotaxic instrument manipulator sizes. By providing the details of the StereoPylot build process and making all of our files open access, we hope that StereoPylot will be a useful resource for the neuroscience research community and encourage users to take advantage of the highly customizable features to accommodate their specific experimental needs.

**Movie 1.** Demonstration of the StereoPylot in use, with examples of what each button does and how to use the offsets to quickly switch between drills and syringe holders. The video also demonstrates how to use the GUI to load custom features such as preset stereotaxic coordinates. [View online]

## Materials and Methods

### Parts list

The materials required for the assembly of the StereoPylot instrument are summarized in Table 1. It is important to note that although some items may come in sets, the total number of each required item is listed in the table, and these numbers do not refer to the number of sets. All relevant 3D object files and code are provided in Extended Data 1.

**Table 1.** List of parts required for the StereoPylot conversion

Item	Number needed	Supplier	Code
PCB board	1	PCBasic	Custom (see Gerber Files)
10 kΩ resistor	24	Digikey	CF14JT10K0CT-ND
100 kΩ resistor	24	Digikey	CF14JT100KCT-ND
Ceramic capacitor (1 uF 50 V)	24	Digikey	399-9886-1-ND
Hex inverter Schmitt trigger (SN74HC14)	4	Digikey	296-1577-5-ND
Shift register (SN74HC165N)	3	Digikey	296-8251-5-ND
IC DIP socket—16 position (2.54 mm Pitch)	7	Digikey	ED3016-ND
Rotary encoder (EC11, vertical, push button)	3	Digikey	EC11E18244AU
Motor driver expansion board (fits A4988 and DRV8825)	3	Amazon	B08RP2SCJ7
A4988 stepper driver	3	Amazon	B0CD2F5K4D
Round button tactile switch (12 × 12 × 7.3)	16	Amazon	B01E38OS7K
Power supply (12 V with 5.5 × 2.5 mm jack)	1	Amazon	B01GEA8PQA
Female power jack 5.5 × 2.5 mm	1	Amazon	B0F28MB721
Raspberry Pi 3B+	1	pishop.ca	9001
Micro SD card (min 16GB)	1	Amazon	B074B4P7KD
3 Position ON/Off/ON Toggle rocker switch	1	Amazon	B085ZPD1FM
Momentary micro limit switch	3	Amazon	B0C8THL9NV
T-type GPIO breakout Expansion board with ribbon cable	1	Amazon	B089SXW3HD
LM2596S DC-DC stepdown buck converter 3A	2	Amazon	B07PCSL919
Waveshare Raspberry Pi 5 Inch LCD HDMI 800 × 480	1	Amazon	B01HPV7D38
Male to up angled 90D HDMI Male HDTV FPC Flat Cable	1	Amazon	B07R6CWPH1
NEMA17 stepper motor	2	Amazon	B0D1QFFHDK
NEMA17 stepper motor (55+ Ncm torque)	1	Amazon	B06ZYQNBFR
PCB solderable breadboard, 5 × 7 cm Universal	1	Amazon	B0BGNR2G31
Universal Board Solderable BreadBoard	3	Amazon	B083YTPYYQ
Expandable Braided Sleeving (0.25″)	1	Amazon	B07S81TNXL
GT2 20 tooth pulley 5 mm bore	1	Amazon	B078Z6YZCY
GT2 20 tooth pulley 4 mm bore	1	Amazon	B078Z7ZGGF
GT2 142 mm closed loop belt 6 mm width (71 teeth)	1	Amazon	B0BZNNV1J9
Brass Shaft Coupler (4 mm to 5 mm Bore Rigid Coupling 20 mm length 9 mm diameter)	3	Amazon	B0D3TKG5WN
Micro-USB Male Type B 5 Terminal Jack Port Solder Connector	1	Amazon	B0BR8V2X1B
Rii i8+ Mini Wireless Touch Keyboard	1	Amazon	B00WQG6A8C
M2 bolts (20 mm) and nuts	12	Amazon	B015A313T8
M5 0.8 pitch tap	1	Amazon	B077HTXVR1
Printable vinyl sticker paper	1	Amazon	B0CJF87KDM
6 inch USB extension	1	Amazon	B00S2N2Q4U
Wire 24 gauge		Amazon	B075M55F7S
Wire 22 gauge		Amazon	B075M7YZXC
Wire 20 gauge		Amazon	B073RDG2J6
Wire 18 gauge		Amazon	B0CPW6WP8S
M2 bolt assortment		Amazon	B0C6M6MJ8M
M3 bolt assortment		Amazon	B0C7ZPZ214
Heat shrink tubing (assorted sizes)		Amazon	B0778D22WM
3D-printed components			
File Name	Number needed		
Control_Box_Lower_Box.stl	1		
Control_Box_Base_Cover.stl	1		
Control_Box_Mid_Box.stl	1		
Control_Box_Cableholder.stl	1		
Control_Box_Upper_Face.stl	1		
Motor_Mount_Left.stl	2		
Motor_Mount_Right.stl	2		
Motor_Mount_Rear.stl	1		
Tapping_Guide.stl	1		

5 × 7 cm breadboard cuts	Number needed		
5 × 7 cm	2		
3 × 3 cm	1		
5 × 4 cm	1		
2.5 × 7 cm	1		
2.5 × 3 cm	1		

Please note that a 3D printer is required for the control box, motor mounts, and tapping guide. 3D models are provided in .STL format which is compatible with a range of commercially available 3D printers.

### Stereotaxic equipment

StereoPylot was designed to be compatible with a variety of stereotaxic instruments that use left or right three-axis manipulators with 0.1 mm resolution scales. While the construction of most 0.1 mm resolution manipulators is similar across vendors, there are minor size differences that affect the fit of the provided 3D-printed motor mounts. We have therefore provided 3D motor mount models for three common 0.1 mm manipulator arm sizes to ensure the compatibility of StereoPylot with a variety of stereotaxic instruments. Fitting the 3D-printed motor mounts on stereotaxic instruments with a significantly different design, such as ultraprecise 0.01 mm resolution manipulators, will require modifications to the design of the CAD file to ensure proper fit.

### 3D Printing

**Figure 3.**
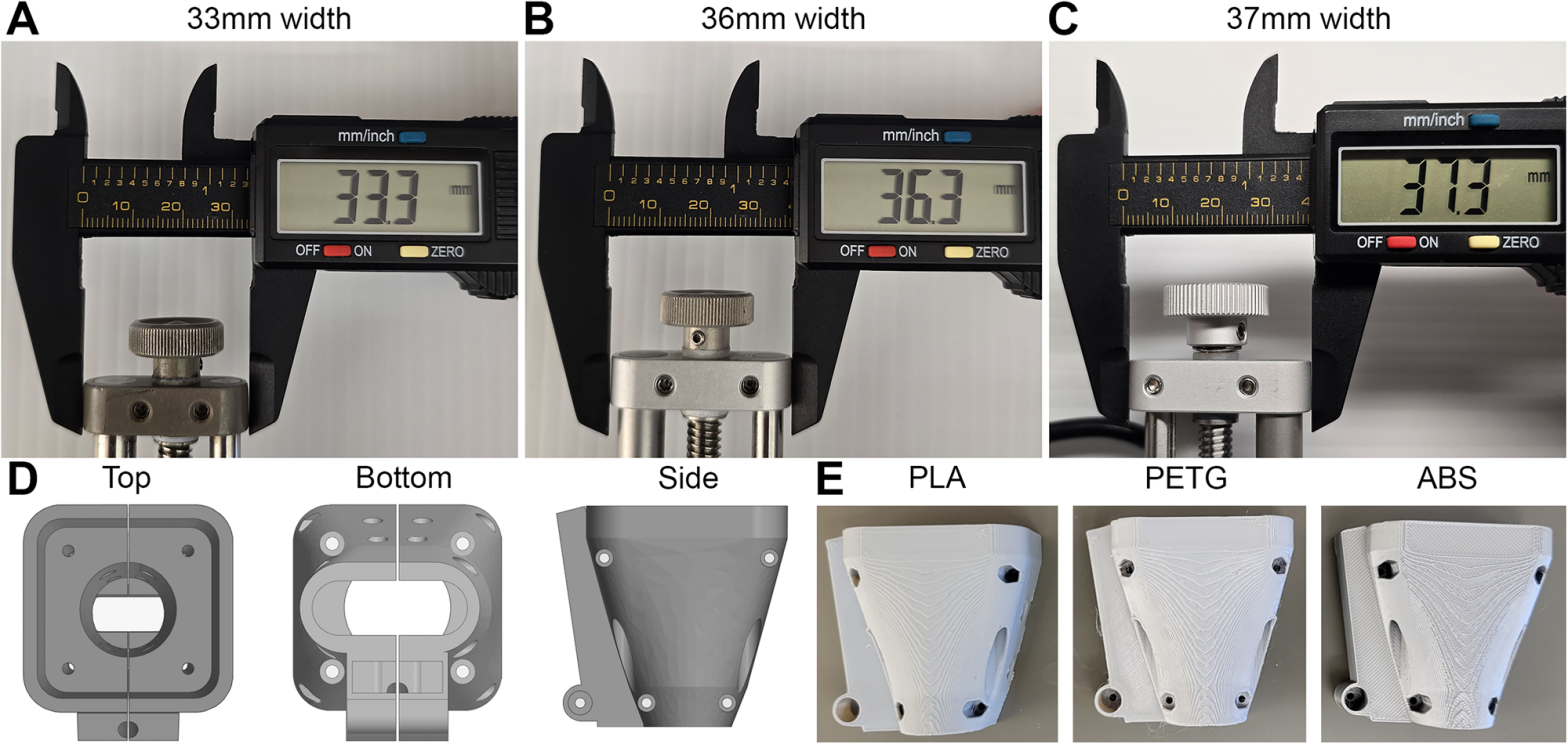
StereoPylot is compatible with a variety of manipulator sizes. Although 0.1 mm resolution manipulators feature a relatively standard design, the physical dimensions of manipulators often differ between manufacturers. We have provided 3D motor mount files for three common manipulator sizes: (***A***) 33 mm wide, (***B***), 36 mm wide, and (***C***) 37 mm wide. To determine which 3D motor mount model is required, use a micrometer to measure the width of the manipulator, as shown. ***D***, Representative 3D render showing the top, bottom, and side profiles of the 3D motor mount file. ***E***, Side profile photos of 3D-printed motor mounts demonstrate that the 3D file is compatible with a variety of printing materials, including PLA, PETG, and ABS. We recommend using PETG or ABS filaments for printing 3D motor mounts due to their superior strength and heat resistance.

Refer to Table 1 for a list of the 3D models and the number of copies required to print. The 3D models were designed using the FreeCAD software and exported in both STL and STEP file formats for maximum compatibility. To determine which motor mount model to 3D print, measure the length of the manipulator end bracket (Fig. 3), round down to the nearest millimeter, and then print the corresponding 3D motor mount files available on our GitHub page (see below, Code accessibility). If the motor mounts require further adjustments, the 3D models can be edited in the CAD software. We have also provided a 3D tapping guide model to facilitate tapping of brass couplers that connect the stepper motors to the manipulator arms (Movie 2). Several 3D model files are also provided to build the StereoPylot control box (lower box, middle box, upper control face, base cover, and cable holder). The 3D models were sliced in UltiMaker Cura software (V 5.11) or Bambu Studio (V 02.02.02.56) using default settings and then printed with an Anycubic Kobra 2 or BambuLab H2S printer, respectively. Standard polylactic acid (PLA) 3D printing filament worked with all 3D model files, but we recommend using polyethylene terephthalate glycol (PETG) or acrylonitrile butadiene styrene (ABS) for the motor mount files since these filaments offer superior strength and heat resistance (Fig. 3). We also recommend that users regularly check the motor mounts and replace them if signs of mechanical wear are present (e.g., cracks).

**Movie 2.** Video showing how to tap a thread in the brass coupler to connect the stepper motor to the stereotaxic manipulator. The set screws are removed from the brass coupler which is then inserted into the 3D-printed tapping guide. A benchtop vice is used to hold the guide and coupler secure during the tapping process. A M5 0.8 pitch tap is inserted into the tap holder and a small amount of light oil is applied to the tap. The tap is aligned with the hole in the guide and turned clockwise. As the tap cuts into the hole, the guide will help to straighten out the tap before it starts cutting the thread into the brass coupler. When the tap has been turned to its full depth, reverse the direction to remove the tap and inspect the newly threaded coupler. [View online]

### Assembly of the upper control box

**Figure 4.**
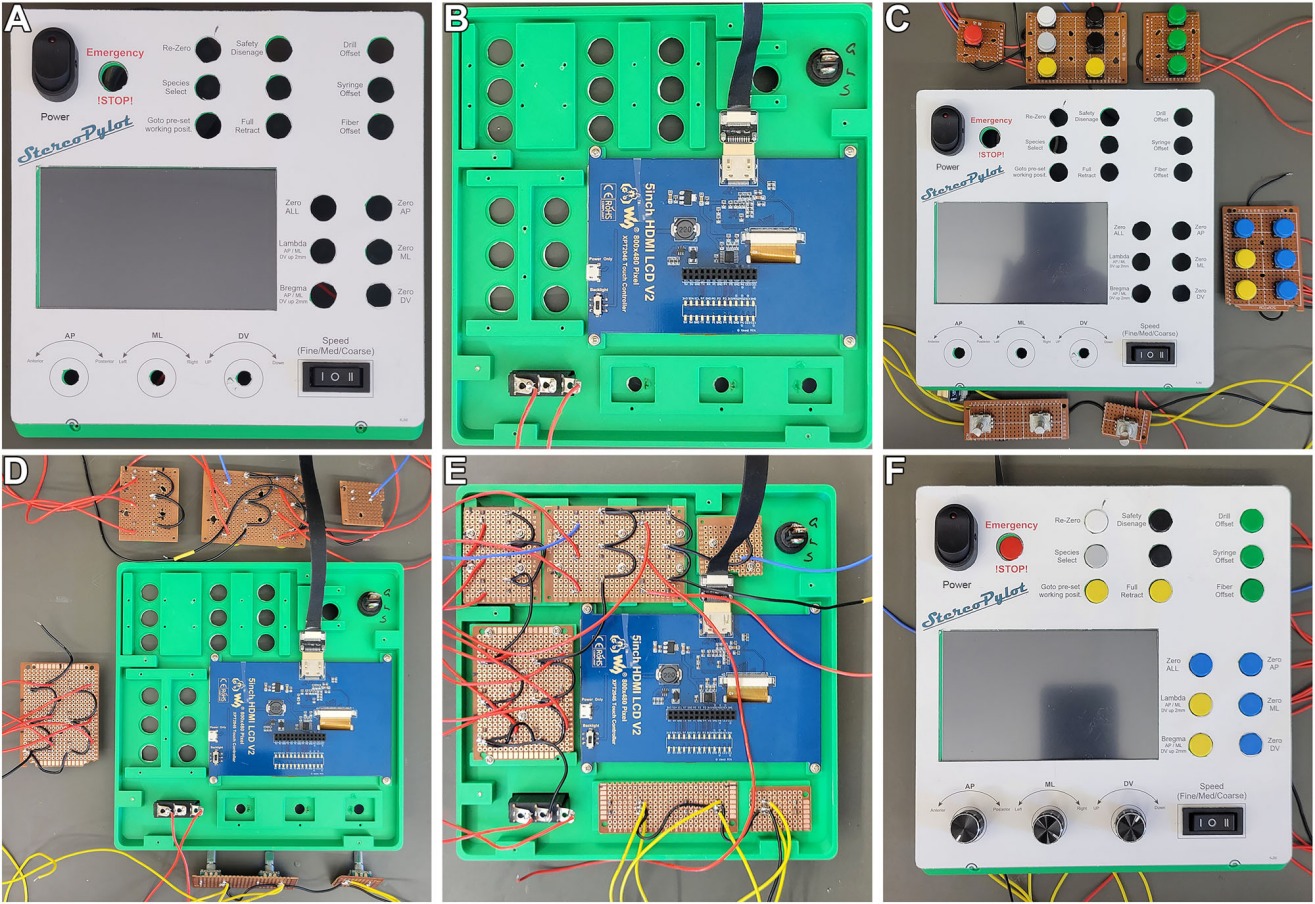
Overview of the StereoPylot control face assembly. ***A***, Upper control box with the button overlay applied and with power and speed selector switches installed. ***B***, The 5 inch LCD screen is installed, and the HDMI ribbon cable is attached. ***C***, Buttons and encoders are positioned on the breadboards so that they align with the holes in the upper control box and are soldered in place. ***D***, Wire leads are soldered to the button data pins, and the ground pins are connected in series. ***E***, Installation of the button boards using M2 bolts and the ground pins between the boards are connected. ***F***, The knobs for the encoders are pressed on to complete the upper control box assembly.

Once all required components are ordered and the 3D model files are printed, construction of the StereoPylot control box can begin. The upper control box was assembled first (Fig. 4). A vinyl button overlay was applied to the face of the upper control box, followed by installation of the power and speed selection switches (Fig. 4*A*). The LCD display was then mounted on the backside of the upper control box and secured with screws (Fig. 4*B*). Tactile buttons were then positioned on breadboards cut to size (Table 1), and preliminary button wiring was completed before mounting the breadboards onto the backside of the upper control box (Fig. 4*C*,*D*). The button breadboards were then secured to the upper control box via M2 bolts (Fig. 4*E*). Once the breadboards were secure, the control box was rotated to the overlay side, and the knobs for the rotary encoders were attached to complete assembly of the upper control box (Fig. 4*F*).

**Figure 5.**
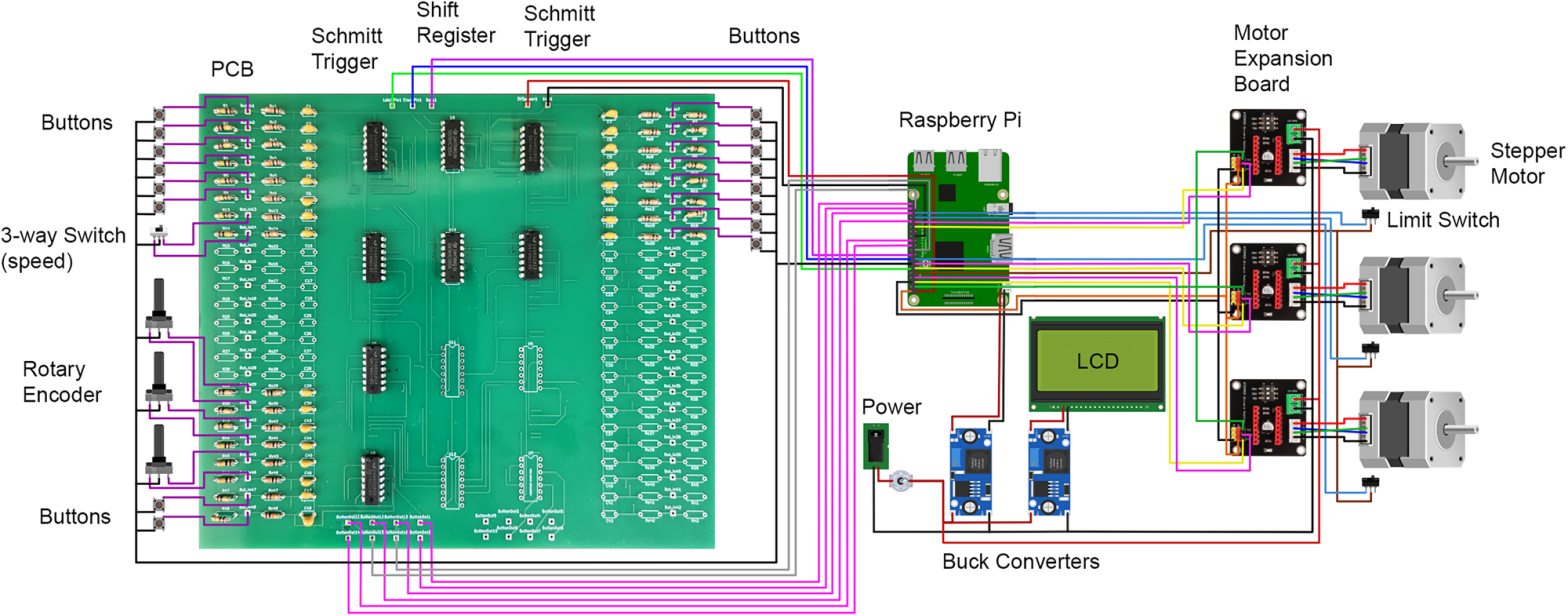
Wiring diagram. Illustration of the custom PCB with the wired connections to the electronic components of StereoPylot mapped out. The diagram shows the location for the Schmitt triggers and shift registers as well as the position of the three groups of resistors and capacitors that connect to the buttons and encoders. The vacant positions on the PCB are available for additional components to support an additional 16 buttons through the shift registers and 8 through direct connections to the Raspberry Pi.

**Figure 6.**
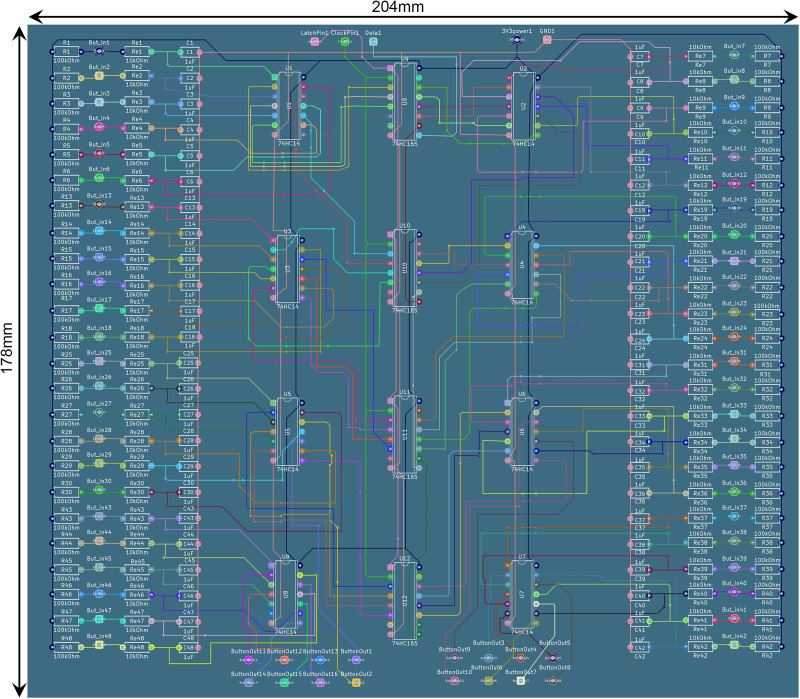
Custom PCB. Full schematic of the custom PCB capable of supporting up to 32 digital inputs via shift registers and 16 through direct connection to the Raspberry Pi. The components are arranged in columns with the shift registers occupying the central column. Schmitt triggers are placed in the columns flanking the shift registers. Moving further outward, there are columns on both sides of the board for 1 uF capacitors, 10 kΩ resistors, and 100 kΩ resistors, respectively. The inputs are connected to soldering pads between the 10 kΩ and 100 kΩ resistors. The top 16 rows of resistors and capacitors are read by the shift registers and the bottom 8 rows connect directly to the Raspberry Pi. The upper edge of the PCB has connection points for 3v3 power and ground wires from Raspberry Pi to energize shift registers and Schmitt triggers. To the left of the 3v3 power connection, there are solder points for latch, clock, and data wires which are used to read the shift registers. The lower edge of the board has two groups of eight solder pads that bypass the shift registers and connect inputs directly to the Raspberry Pi.

The wiring of the StereoPylot was done in sequential stages as various components of the control box were assembled. An illustrated drawing of the wiring was generated with Fritzing software (https://www.fritzing.org) to provide a guide for the assembly process (Fig. 5). To assist with the assembly of StereoPylot, we designed a custom-printed circuit board (PCB) to simplify the installation of the electrical components. A schematic of the PCB is provided in Figure 6. StereoPylot has 15 buttons, a three-way switch, and three rotary encoders. Notably, all inputs are debounced using an R-C circuit combined with a Schmitt Trigger (DigiKey, #296-1577-5-ND) which filters and smooths the output when switching states. The debounced output from the encoders and two of the buttons are connected directly to the Raspberry Pi data pins to allow for instantaneous, event-driven control. To accommodate the remaining buttons and three-way switch, we used shift registers (DigiKey, #296-8251-5-ND) which can receive debounced inputs from many sources and relay the data while using only three input pins on the Raspberry Pi (see Table 2 for complete map of the Raspberry Pi connections). A full wiring schematic is included in our GitHub repository (see below, Code accessibility). To promote engagement and further development of StereoPylot, the PCB is designed to accept up to 48 inputs: 16 that output directly the Raspberry Pi and 32 through shift registers. New functionality can be implemented with additional buttons and edits to the software code. A complete description of the installation of the electronic components for StereoPylot and the Raspberry Pi setup is provided in the supplemental materials (Extended Data 2).

**Table 2.** Raspberry Pi Pin layout

Connection	GPIO	Side with SD card slot			
		Pin number	Pin number	GPIO	Connection
	3v3	1	2	5v	stepper 5V
Enable ALL	2	3	4	5v	
dir AP	3	5	6	GND	stepper GND
step AP	4	7	8	14	
Limit GND	GND	9	10	15	
dir ML	17	11	12	18	LatchPin
step ML	27	13	14	GND	Button GND
limit AP	22	15	16	23	ClockPin
PCB Board power	3v3	17	18	24	DataPin
Safety Button	10	19	20	GND	
	9	21	22	25	encoderAP_A
	11	23	24	8	encoderAP_B
Button Board GND	GND	25	26	7	
	0	27	28	1	
dir DV	5	29	30	GND	
step DV	6	31	32	12	encoderML_A
limit ML	13	33	34	GND	
limit DV	19	35	36	16	encoderML_B
EMERG STOP	26	37	38	20	encoderDV_A
	GND	39	40	21	encoderDV_B

### Power supply management

**Figure 7.**
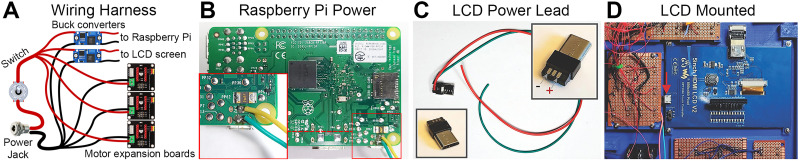
Overview of power supply management. The stepper motors require 12 V whereas the LCD and Raspberry Pi depend on 5 V power supply. The unit receives power from a 12 V supply, so a wiring harness needs to be made. ***A***, An illustration of the wiring harness showing 18 gauge wire connecting the positive central terminal of the power jack to the supply side of a single pole single throw (SPST) power switch. A second length of 18 gauge wire is connected to the load side of the SPST switch, and, to that wire, five lengths of 20 gauge wire are soldered. Two of these 20 gauge wires connect to the IN + terminals of the buck converters; the other three are connected to the positive terminals of the motor expansion boards. The 20 gauge ground wires from the IN − terminals of the buck converters and from the motor expansion boards are all connected to a length of 18 gauge wire which is then soldered to the outer ground terminal of the power supply jack. The buck converters are adjusted to 5.15 V to provide power to the Raspberry Pi and the LCD screen. ***B***, Test points PP2 (+) and PP5 (−) on the Raspberry Pi are used to power the unit. These test points are located to the lower right of the SD card slot on the back of the Raspberry Pi. A magnified view (inset) shows the test points with a yellow lead soldered to the PP2 and a green lead to PP5. The free ends of the wire are soldered to one of the buck converters preset to 5.15 V with PP2 going to the positive terminal and PP5 to the negative. ***C***, A male micro-USB jack with positive and negative leads attached will be used to power the LCD screen through the power port on the side of the screen. Two leads need to be soldered to a male micro-USB jack (pictured in bottom-left inset). The top-right inset shows the solder points with the location of the positive and negative terminals. The free end of the leads will be soldered to a buck converter preset to 5.15 V. ***D***, The LCD screen is mounted to the faceplate by four 4 mm M2 bolts with washers. The red arrow shows the location of the micro-USB port. The adjacent button board may have to be temporarily loosened so that the micro-USB jack can slide underneath.

A 12 V power supply was used to power the stepper motors, LCD screen, and Raspberry Pi. A wiring harness was fabricated to distribute power from a single source to the individual components (Fig. 7*A*). To power the Raspberry Pi, the 12 V power source was stepped down to 5.1 V using a buck converter (Amazon, #B07PCSL919) and hard wired to the solder points under the USB power port on the Raspberry Pi (Fig. 7*B*). A second buck converter was used to step down the 12 V power to 5.1 V and connected to a micro-USB male jack (Amazon, #B0BR8V2X1B; Fig. 7*C*) which was then plugged into the power port of the LCD screen (Fig. 7*D*).

### Assembly of the lower control box

**Figure 8.**
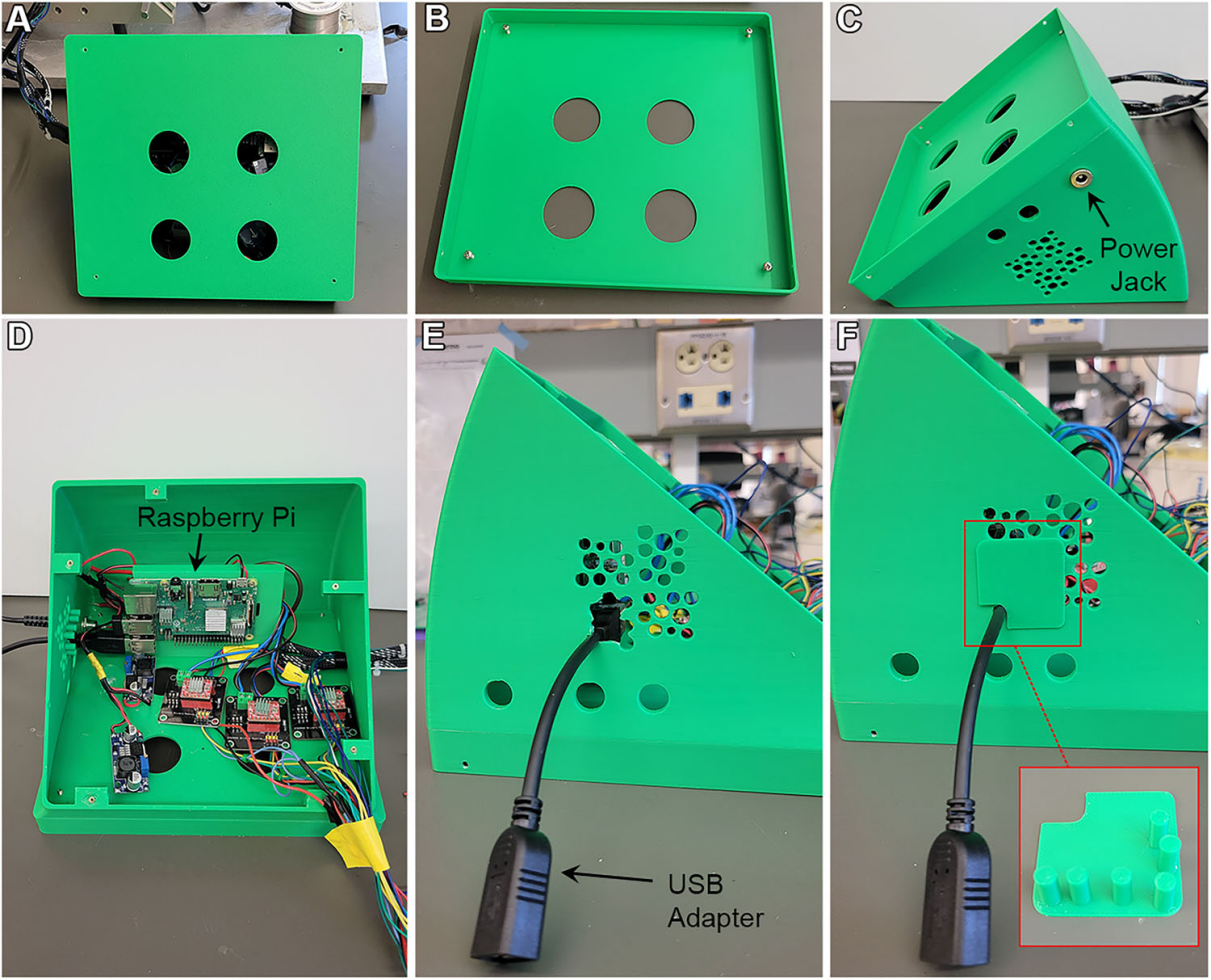
Assembly of the lower control box. ***A***, The middle control box has four mounting holes in each corner and four large wire passthrough holes. ***B, C***, Four 6 mm M3 bolts are started in the corner holes of the 3D-printed lower control box and then attached to the bottom of the middle control box and the power supply jack is installed on the right side. ***D***, The Raspberry Pi is mounted, and the wiring harness is routed to position the motor expansion boards and buck converters. ***E***, The USB adapter cable is connected to the Raspberry Pi and routed through a hole which is cut in the vent. ***F***, The 3D-printed cable holder covers the hole in vents and secures the USB adapter cable.

The 3D-printed mid box (Fig. 8*A*) was secured to the lower control box (Fig. 8*B*) to form a single unit. We then installed a power jack (Amazon, #B01GEA8PQA) in the rear left port of the lower control box (Fig. 8*C*), followed by installation of the wiring harness, Raspberry Pi, motor expansion boards, and buck converters (Fig. 8*D*). Next, we connected a USB extension adapter cable (Amazon, #B00S2N2Q4U) to the Raspberry Pi to facilitate offline file transfers. A hole was cut in the left side ventilation ports to allow the USB adapter cable to pass through the lower control box wall (Fig. 8*E*). This hole was then covered with a 3D-printed patch designed to secure the USB adapter in place (Fig. 8*F*).

### Adding stepper motors to the micromanipulators

**Figure 9.**
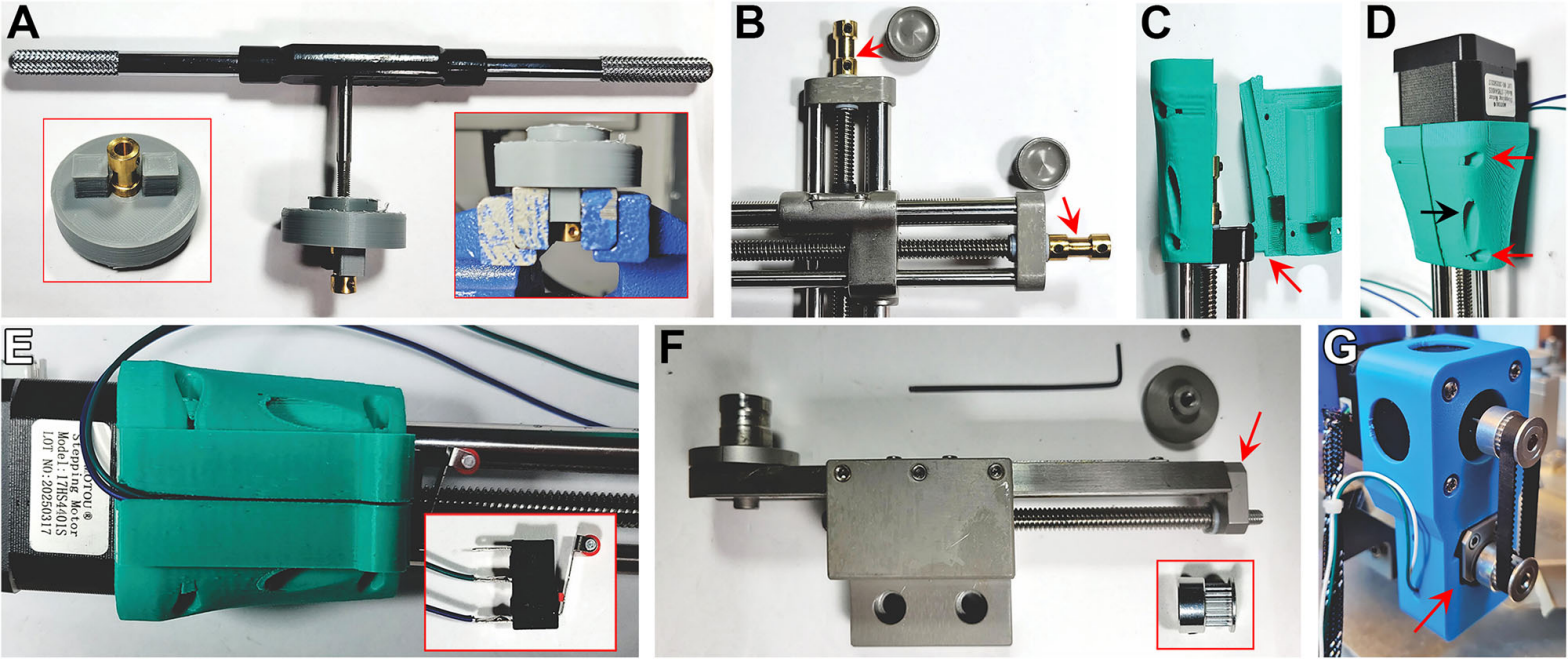
Attaching the stepper motors to the stereotaxic manipulators. ***A***, The brass couplers need to be tapped with a M5 0.8 pitch tap. The 3D-printed tapping guide is included in the model files to facilitate a straight tap (left inset). A vice holds the coupler and the tapping guide while tapping the threads (right inset). Take care to avoid tapping at an angle or overtightening, as this can strip the bore. ***B***, The set screws of the upper manipulator knobs are then loosened so the knobs can screw off. The threaded brass couplers are screwed on and secured with the set screws (red arrows). ***C***, Test fitting the motor mounts. The red arrow indicates where a thin strip of electrical tape is to be applied to make a firm fit. With one-half of the mount in place (as shown) fit the stepper shaft into the brass coupler and secure the set screws. ***D***, Use four 25 mm M2 bolts with nuts to secure the left and right motor mounts together. Ensure that the fit is snug but avoid overtightening as this can cause twisting and binding. The red arrows show the location of the M2 bolt holes on one side, there are two more on the opposite side. The black arrow shows the location of the M3 bolt hole to secure the motor, use two bolts on opposite corners. ***E***, The limit switch wires are threaded through the channel on the side of the motor mount, and the switch is recessed in the mount. The insert shows a switch wired in the normally open position. ***F***, The knob of the lower manipulator is removed in the same fashion as the upper manipulators and the rear faceplate (red arrow) is removed as well. The 4 mm bore 20-tooth gear (inset) needs to be tapped with M5 0.8 pitch tap. ***G***, Picture of the assembled lower manipulator motor. The red arrow shows the face plate that was reattached after sliding the mount in place and the threaded gear that is installed afterwards.

Once the control box was mostly assembled, we attached stepper motors to the drive shaft of the ML and DV manipulator arms via brass couplers (Amazon, #B0D3TKG5WN). We used a 3D-printed tapping guide to tap threads into one end of the brass coupler (Fig. 9*A*; Movie 2), which were then turned onto the manipulator arm drive shaft and secured with set screws (Fig. 9*B*). The 3D-printed motor mounts were test fitted to ensure a snug fit (Fig. 9*C*), and then the stepper motor was connected to the coupler and enclosed within the motor mount (Fig. 9*D*). Limit switches were secured into recesses within the motor mounts, and the wires were routed through a channel in the 3D motor mount (Fig. 9*E*). Next, the adjustment knob and rear plate of the anterior–posterior (AP) manipulator was removed, the threads were tapped in the pulley (Amazon, #B078Z7ZGGF), which were then turned onto the drive shaft of the manipulator (Fig. 9*F*). The rear motor mount and stepper were installed using the previously removed rear plate to secure the mount. A second pulley (Amazon, #B078Z6YZCY) was secured to the stepper motor and fitted with a 6-mm-wide timing belt (Fig. 9*G*). The stepper motor and limit switch wires were then routed into the lower control box and connected to the stepper motor expansion boards (Amazon, #B08RP2SCJ7).

**Figure 10.**
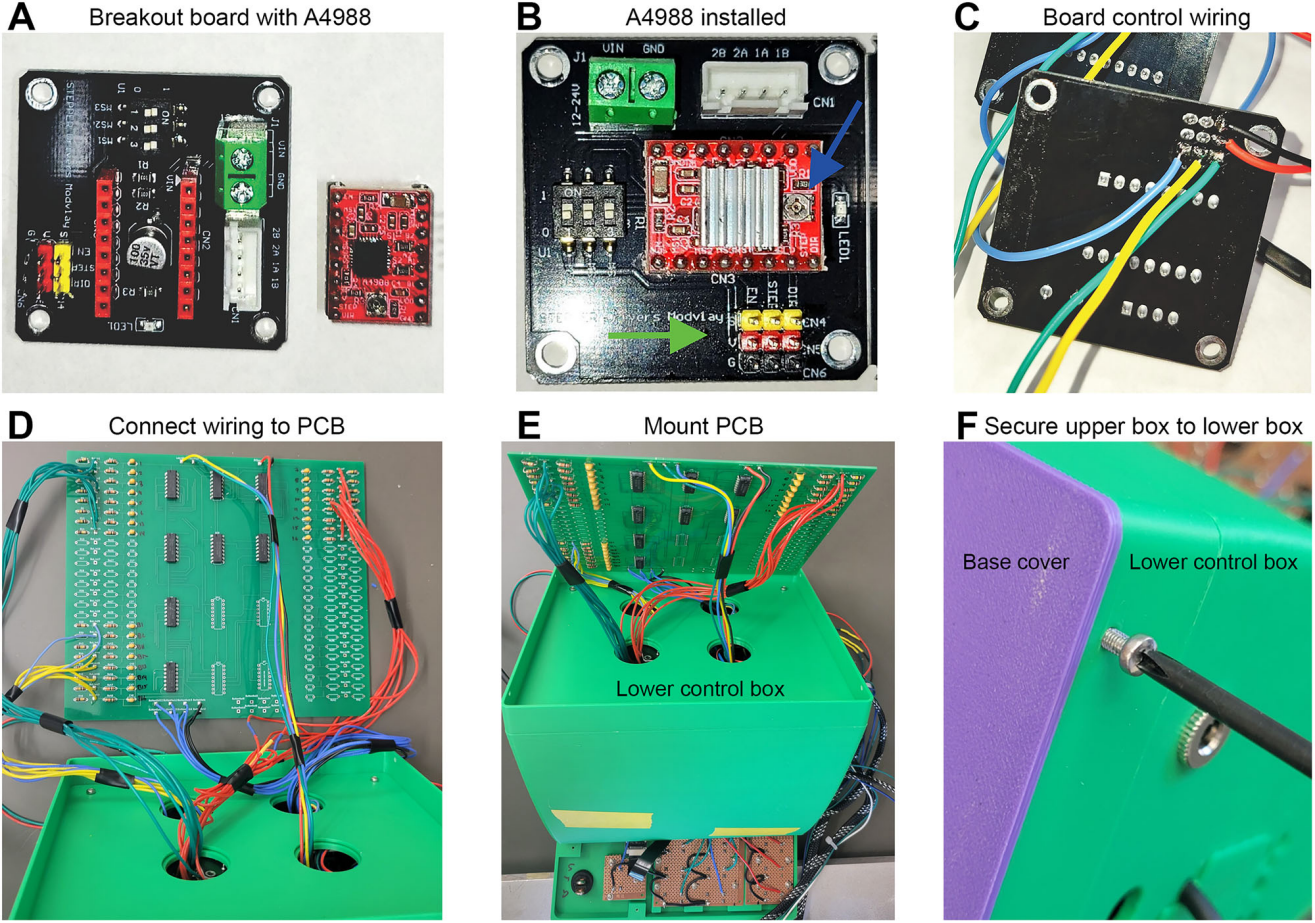
Setup of the stepper motor drivers and final installation of the control box. ***A***, We used the A4988 stepper with the breakout board for the final build. The stepper driver inserts into the header pins on the breakout board, so it is easily replaced. It also provides the option to use the DRV8825 stepper which can support higher voltage and current. The green port on the top right receives 12 V power and the white port connects to the steppers. ***B***, The A4988 needs to be installed in the correct orientation. The resistor potentiometer (blue arrow) faces the edge of the board and must be adjusted to provide enough current to operate the motors. The green arrow shows the Raspberry Pi pin connections with red 5 V voltage input and black grounding pins. Also shown are the pins in the yellow header which have the Enable, Step, and Dir pins that connect to the Raspberry Pi. The black box (middle left) contains the switch to adjust the microstepping. ***C***, Example of the wiring for the breakout board. Each axis requires a board and a driver. All three boards are connected to the same 5 V power pin on the Raspberry Pi (red wire); the same applies to the ground pin (black wire) and the Enable pin (blue wire). The Step (yellow wire) and Dir (green wire) pins each have their own pin on the Raspberry Pi. ***D***, The completed wiring is then routed from the upper control box and connected to the PCB. ***E***, Once wiring of the PCB is complete, the PCB is then placed in the recessed area of the lower control box. ***F***, The upper control box is then secured to the lower control box to complete assembly of the StereoPylot controller.

The stepper motors are controlled by A4988 stepper drivers connected to breakout boards since we found that this approach simplified the communication between the Raspberry Pi and stepper drivers (Fig. 10*A*). The current output of the stepper drivers was adjusted by carefully turning the resistor potentiometer (Fig. 10*B*, blue arrow). The stepper resolution of the motors can be adjusted from 1/16 to a full step by adjusting the MS1, MS2, and MS3 switches to the left of the A4988 driver (Fig. 10*B*). In our testing, we have found that the half step resolution provides the best balance of speed and accuracy, but users can adjust the stepper resolution for their specific use cases (Table 3). The power and Raspberry Pi control wires can then be soldered in place (Fig. 10*C*). Once the stepper motor wiring was completed, the preliminary button wires from the upper control box were routed through the bottom of the lower box and connected to the PCB inputs (Fig. 10*D*). The PCB wiring was completed by connecting the power supply and outputs of the PCB to Raspberry Pi (Fig. 10*E*). The upper control box was then secured to the lower control box to complete the construction of the StereoPylot controller (Fig. 10*F*). Please refer to the Extended Data 2 for complete details.

**Table 3.** Position of the MS1, MS2, and MS3 switches

MS1	MS2	MS3	Microstep resolution
Off	Off	Off	Full step
On	Off	Off	1/2 step
Off	On	Off	1/4 step
On	On	Off	1/8 step
On	On	On	1/16 step

### Initial StereoPylot calibration

Once the StereoPylot control box is built and all stepper motors are installed, StereoPylot is ready to be powered on. On startup, the software will ask the user if a calibration step is required. The software will prompt the user to perform an initial reading using the vernier scale on the manipulator axis and enter the value. The software will advance a predefined number of steps and then will ask the user to read the vernier scale again and enter the final position reading. This process will be performed for each axis, and the software will use the entered values to calculate the distance traveled per stepper motor step.

### Accuracy and precision of StereoPylot

**Figure 11.**
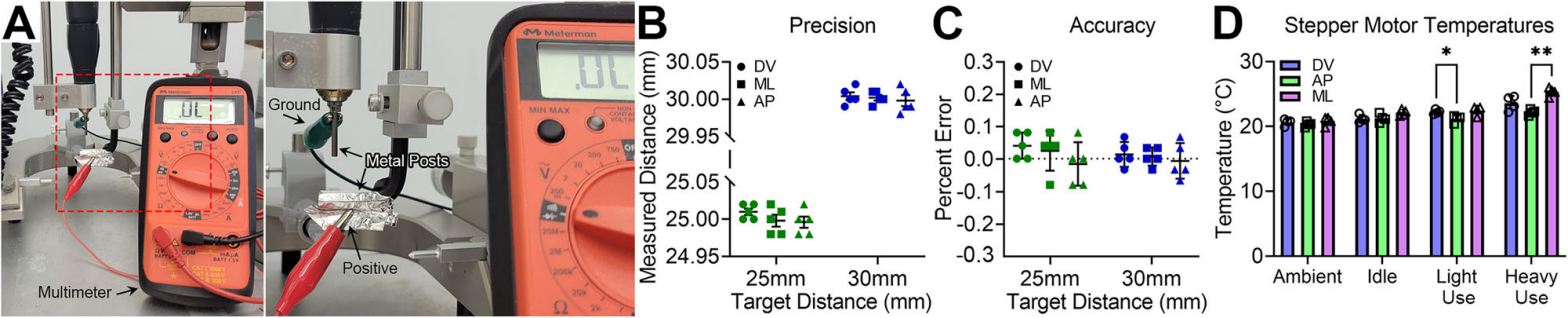
Validation of StereoPylot precision, accuracy, and operating temperatures. ***A***, To measure the accuracy and precision of StereoPylot, a manual manipulator with 0.01 mm accuracy was installed opposite to the StereoPylot manipulator and a probe holder was used to hold an Allen key in position. A multimeter was set to test continuity by connecting the positive and ground terminals to the metal drill bit and Allen key. Whenever the metal drill bit and Allen key touched, the circuit would complete and the multimeter would emit an audible beep. ***B***, Precision, the repeatability of the measurements, was measured by calculating the standard deviation of the measurements after traversing either 25 or 30 mm. The maximum standard deviation across all axes was 0.016 mm. ***C***, Accuracy, the closeness of the measurements to the intended target, was expressed as percent error. The maximum percent error across all three axes was 0.08% or ∼0.02 mm. ***D***, The ambient temperature of the stepper motors was taken before powering on StereoPylot. Temperature readings were taken again immediately after initial setup and calibration while idle and again after light and heavy use. Light use corresponds to typical movements experienced during surgery whereas heavy use consists of multiple, continuous, back and forth movements of 35 mm. All graphs depict mean ± SD. *p < 0.05; **p < 0.01.

Once the calibration step was completed, we sought to determine the accuracy and precision of the StereoPylot instrument. An ultraprecise manipulator (KOPF Model 962 series) was attached to the arm opposite the StereoPylot manipulator, and an Allen key was secured to a syringe holder. The lower end of the Allen key was wrapped with tinfoil, and the negative lead of a digital multimeter was connected to the foil; the positive lead was connected to the metal probe held by the drill attached to the StereoPylot manipulator. The multimeter was set to measure continuity so that when the drill bit and the Allen key came into contact, the multimeter would emit an audible beep to indicate a complete circuit (Fig. 11*A*). To measure the movement of the StereoPylot manipulator, the two manipulators were adjusted so that the multimeter registered a complete circuit. The position of the ultraprecise manipulator was then recorded. The StereoPylot manipulator was then moved a set distance, and the ultraprecise manipulator was adjusted until the multimeter registered a complete circuit again. This procedure was repeated five times with 25 and 30 mm movements in each axis. We calculated the accuracy of the StereoPylot, defined by how close the measured value was to the target value, by using the following formula: Percent Error(Accuracy) = [(Measured Value − True Value) / True Value] × 100. Precision, which indicates how close the measures of multiple movements are to each other, was calculated as the standard deviation of the measured values. The more precise a machine, the lower the variability between measured movements and therefore a lower standard deviation.

### StereoPylot operating temperatures

We also evaluated the thermal performance of StereoPylot by taking temperature recordings of the stepper motors with an infrared thermometer (Inkbird, #INK-IFT01). The backside of each stepper motor was scanned with the infrared thermometer, and the maximum temperature was recorded. Ambient temperature readings were taken prior to turning on StereoPylot. Additional readings were taken at idle, under light use and after heavy use. Idle readings were taken immediately after powering on StereoPylot and completing the initial zeroing and calibration procedure. To obtain readings under light use, temperature recordings were taken after performing movements that were comparable to a standard rodent stereotaxic surgery. Over a 5 min period, each axis was moved 15 mm in each direction five times using the coordinate feature of StereoPylot, followed by five 15 mm movements of each axis using manual rotary knobs. Heavy use readings were taken after using the coordinate feature to perform continuous, repeated movements of 35 mm for 10 min. Four sets of readings were taken with a 1 h interval between readings to allow the stepper motors to reach ambient room temperature between measurements.

### Animals

Adult male and female Long–Evans rats (2–4 months old) were ordered from Charles River Laboratories and pair housed in a vivarium (12 h light/dark cycle, lights on at 7:00A.M.). Rats were housed in standard ventilated cages with a plastic tube for environmental enrichment and food and water provided *ad libitum*. All procedures followed guidelines established by the Canadian Council on Animal Care and were approved by the University of Saskatchewan Animal Research Ethics Board.

### Stereotaxic surgery

Our laboratory is currently using StereoPylot for several ongoing studies that require stereotaxic surgery. We selected a random subset of rat brains from an ongoing fiber photometry project to quantify the accuracy of StereoPylot viral injections and optical fiber probe implants. Briefly, adult male and female rats (*n* = 3 per sex) underwent stereotaxic surgery using established procedures. Rats were anesthetized with 5% isoflurane, their heads were shaved and then secured to the StereoPylot instrument using ear bars. Tear gel (Bausch + Lomb) was applied to each eye, and the head of each rat was swabbed with chlorhexidine gluconate solution, followed by a subcutaneous injection of slow-release buprenorphine (0.8 mg/kg). Body temperature was maintained at 37°C via a homeothermic blanket with a rectal probe (Harvard Apparatus). Heart rate and blood oxygen saturation were monitored throughout surgery using a pulse oximeter. The scalp was then cut with a scalpel, and the connective tissue was exposed to expose the dorsal surface of the skull and cleaned with sterile phosphate-buffered saline (PBS), pH 7.4. A sterile cotton tip applicator was briefly submerged in 30% hydrogen peroxide and gently applied to the skull surface to help identify bregma.

Using bregma as a reference point, StereoPylot was used to make a craniotomy over the nucleus accumbens (NAc; +1.60 mm AP, −1.80 mm ML). An adeno-associated virus (AAV) encoding the dopamine biosensor GRAB-DA (AAV-hSyn-GRAB-gDA3m, 7.9 × 10^12^ vg/ml, #3265-AAV5, Canadian Neurophotonics Platform Viral Vector Core) was loaded into a 1.0 µl Neuros Syringe (#7001 KH, 32 g, Point Style 4, Hamilton) that was attached to a probe holder mounted on the stereotaxic instrument. The syringe was lowered 7.2 mm ventral from the skull surface to reach the NAc and 0.65 µl of virus was injected at a rate of 0.20 µl/min. The needle remained in place for 1 min and was then lifted 0.1 mm and allowed to rest for an additional 4 min. A 200-µm-diameter optic fiber probe was then lowered into the same location and secured to the skull with jeweler screws and dental cement. Each rat was then placed into a clean cage on top of a heating pad until ambulatory. Rats were monitored daily following surgery and underwent fiber photometry recordings at least 3 weeks later. Upon completing the fiber photometry experiments, rats were deeply anesthetized and perfused with cold PBS followed by 4% formaldehyde fixative. Brains were extracted and stored in 4% formaldehyde fixative for 24 h at 4°C and then transferred to a container with 0.1 M PBS and 0.1% sodium azide until use.

### Quantification of viral expression and optical fiber probe placements

Brains were cut at 50 µm in the coronal plane on a vibratome (#VT1000S, Leica) and stored in a 60% glycerol cryoprotectant solution until use. Brain sections containing the NAc were then rinsed with 0.1 M PBS, counterstained with Hoechst 33342 (1:1,000), and mounted onto coverslips. Whole tile scans of each section were acquired with 2.5× and 10× objectives on a Zeiss Axio Observer 7 widefield microscope equipped with the Zen Software (v. 3.9.101.05000). We used the spline contour tool in the Zen Software to draw the boundaries around the area of viral expression (in square millimeter) for each section, which we refer to as viral vector clouds. We also used the line tool in the Zen Software to measure the distance from the top of the cortex to the top, center, and bottom location of each viral vector cloud. Lastly, we also used the line tool to measure the distance from the top of the cortex to the bottom of the optical fiber probe track. We analyzed three sections per rat and calculated the average value of each measure of interest for statistical analyses.

### Statistics

All statistical analyses were conducted using the Prism software (v 9.5.1, GraphPad) with statistical significance set at *p* < 0.05 for all analyses. Precision, accuracy, and temperature data are presented as mean ± standard deviation. The viral vector data are presented as mean ± standard error of the mean. A two-way ANOVA was used for statistical comparisons of precision, accuracy, temperature, and vector distances, followed by Sidak's or Tukey's post hoc test when appropriate. The vector area data were analyzed with an unpaired *t* test.

### Code accessibility

The code and software described in the paper is freely available online at https://github.com/botterillneurolab/StereoPylot

10.1523/ENEURO.0460-25.2026.d1 Data 1 All relevant code and files for StereoPylot. Download Data 1, ZIP file.

10.1523/ENEURO.0460-25.2026.d2 Data 2 This document provides additional technical details for the StereoPylot build process. Download Data 2, DOCX file.

## Results

StereoPylot is a fully motorized digital stereotaxic instrument built with a mixture of commercially available and 3D-printed components. At the heart of StereoPylot is a Raspberry Pi device that controls all hardware and is connected to a large LCD display with a custom GUI. StereoPylot provides 3D motor mount files in a variety of sizes to ensure compatibility with several 0.1 mm resolution manipulators offered by manufacturers. The stepper motors provide a movement resolution of ∼7.5 μm per step, ensuring that StereoPylot offers highly accurate and precise movements that are comparable to commercially available motorized units. StereoPylot also offers several unique quality of life features, including the ability to toggle between three movement speeds, programmable function buttons, tool offset buttons, optional cloud synchronization, a built-in calibration function, and the ability to manually enter coordinates or load premade target lists for common regions of interest. The control box USB adapter was added to allow users to operate the StereoPylot completely offline once the initial Raspberry Pi setup is complete. Moreover, our custom PCB and open-source design supports further customization as desired. For example, Table 4 provides a summary of edits that can be made to the StereoPylot code to modify functionality.

**Table 4.** Summary of line numbers and edits that can be made to the StereoPylot code

Line number	Description	Edits
33	Button array variable	Using the button positions noted during the test-buttons.py test put the labels in order
38	Button variable list	For each button enter the position number as determined in the test-buttons.py (start at 0)
55	Hard wired buttons	Adjust the reported numbers to match the correct function for each hard wired button
113	Preset working position	Number of steps from zero for each axis to reach a comfortable starting position
123	Stepper control pins	Pins are in pairs if the axis is switched then adjusting the pairs of numbers will correct this
135	Limit switch pins	Adjust the numbers so that the limit switches being activated match what is reported
143	Rotary encoder pins	Adjust the numbers so that the rotation of the encoders matches what is reported
161	Stepper direction	Adjust in pairs if the axis moves in the wrong direction during calibration switch the 1 and 0 for that pair

### Validation of StereoPylot precision, accuracy, and operating temperatures

To determine the precision of the StereoPylot instrument, we compared the measured distance against the target distance of 25 or 30 mm for each axis (Fig. 11*B*). A two-way ANOVA revealed a main effect of distance (*F*_(1,8)_ = 1,236,264; *p* < 0.0001), attributable to differences in measurements between the 25 and 30 mm target distances. However, we observed no significant main effect of axis measurement (*F*_(2,16)_ = 1.22; *p* = 0.32), and no significant distance by axis interaction (*F*_(2,16)_ = 0.32; *p* = 0.73). Sidak's post hoc analysis revealed that there were no significant differences between the DV, AP, or ML axis measurements at 25 or 30 mm target distances (all *p* > 0.39). For 25 mm target distance, the measured mean values ± SD were as follows: DV (25.01 ± 0.01 mm), ML (24.998 ± 0.018 mm), and AP (24.996 ± 0.017 mm). Similarly, at the 30 mm target distance, our measured mean values ± SD were as follows: DV (30.004 ± 0.011 mm), ML (30.002 ± 0.008 mm), and AP (29.998 ± 0.016 mm). Overall, these results suggest that the StereoPylot deviated from the target region <0.016 mm even when traversing distances that significantly exceed normal working distances used for rodent stereotaxic surgeries.

Next, we evaluated the accuracy of the StereoPylot by measuring the closeness of the measurements to the intended target distance, expressed as percent error (Fig. 11*C*). A two-way ANOVA revealed no significant main effect of distance (*F*_(1,8)_ = 0.72; *p* = 0.42), axis (*F*_(2,16)_ = 1.25; *p* = 0.31) and no significant distance by axis interaction (*F*_(2,16)_ = 0.29; *p* = 0.75). Notably, not a single axis measurement exceeded 0.08 percent error, which translates to missing the 25 or 30 mm target distance by ≤0.02 mm. Additional evidence of the accuracy and precision of the StereoPylot instrument is provided in the video files (Movies 3, 4).

**Movie 3.** Video showing the accuracy of the preset stereotaxic coordinates feature in StereoPylot. A 3D-printed mouse skull was mounted onto an ultraprecise stereotaxic instrument via ear bars. We used a permanent marker to label bregma, area CA2 in the left hippocampus, and the dentate gyrus in the right hippocampus. Next, we mounted the 3D-printed skull onto the StereoPylot instrument and attached a surgical drill. We calibrated StereoPylot using bregma coordinates and then used the preset coordinates in the GUI to move the drill over the left hippocampal CA2 and right dentate gyrus to make craniotomies. Both craniotomies successfully drilled through the permanent marker labels that were made on the ultraprecise stereotaxic instrument, demonstrating the high degree of accuracy of the StereoPylot instrument. [View online]

**Movie 4.** Video that demonstrates the precision of the StereoPylot coordinate feature. A drill bit was zeroed to bregma of a 3D-printed mouse skull that was mounted onto an ultraprecise stereotaxic instrument via ear bars. The surface of the model skull was painted black for better visualization. The coordinate feature of the StereoPylot was used to navigate to AP −2.3 mm and ML +1.45 mm, and the drill was lowered manually to drill a hole in the model. The drill was retracted and the coordinate feature was used to manipulate the drill to 25 random coordinates in all three axes. After the 25th movement, the coordinate feature was used to return to AP −2.3 mm and ML +1.45 mm, and the drill was manually lowered to demonstrate the drill bit entering the hole that was drilled at the beginning of the demonstration. [View online]

Lastly, a two-way ANOVA was used to measure the temperature of the StereoPylot stepper motors across a variety of use scenarios (ambient, idle, light use, heavy use; Fig. 11*D*). A two-way ANOVA revealed a significant main effect of axis (*F*_(2,9)_ = 7.52; *p* = 0.01), a significant main effect of load (*F*_(3,27)_ = 114.5; *p* < 0.0001), and a significant axis by load interaction (*F*_(6,27)_ = 9.02; *p* < 0.0001). While idle, the average temperature (21.51 ± 0.12°C) across all stepper motors was one degree higher than the ambient temperature (20.59 ± 0.16°C). The average temperature increased slightly under light use (21.97 ± 0.22°C) and was highest under heavy use (23.68 ± 0.44°C). Tukey's post hoc test revealed that during light use, the DV axis stepper motor (22.25 ± 0.34°C) was ∼1°C warmer than the AP axis stepper motor (21.20 ± 0.54°C; *p* = 0.048). Moreover, during heavy use, the ML axis stepper motor (25.30 ± 0.67°C) was ∼3°C warmer than the AP axis stepper motor (22.18 ± 0.46°C; *p* = 0.001). Taken together, these data demonstrate that even under full load, the maximum operating temperatures of the stepper motors (∼25°C) are well below the heat tolerance ranges for PLA, PETG, and ABS filaments (∼60–100°C).

### Validation of viral vector expression and probe placements

**Figure 12.**
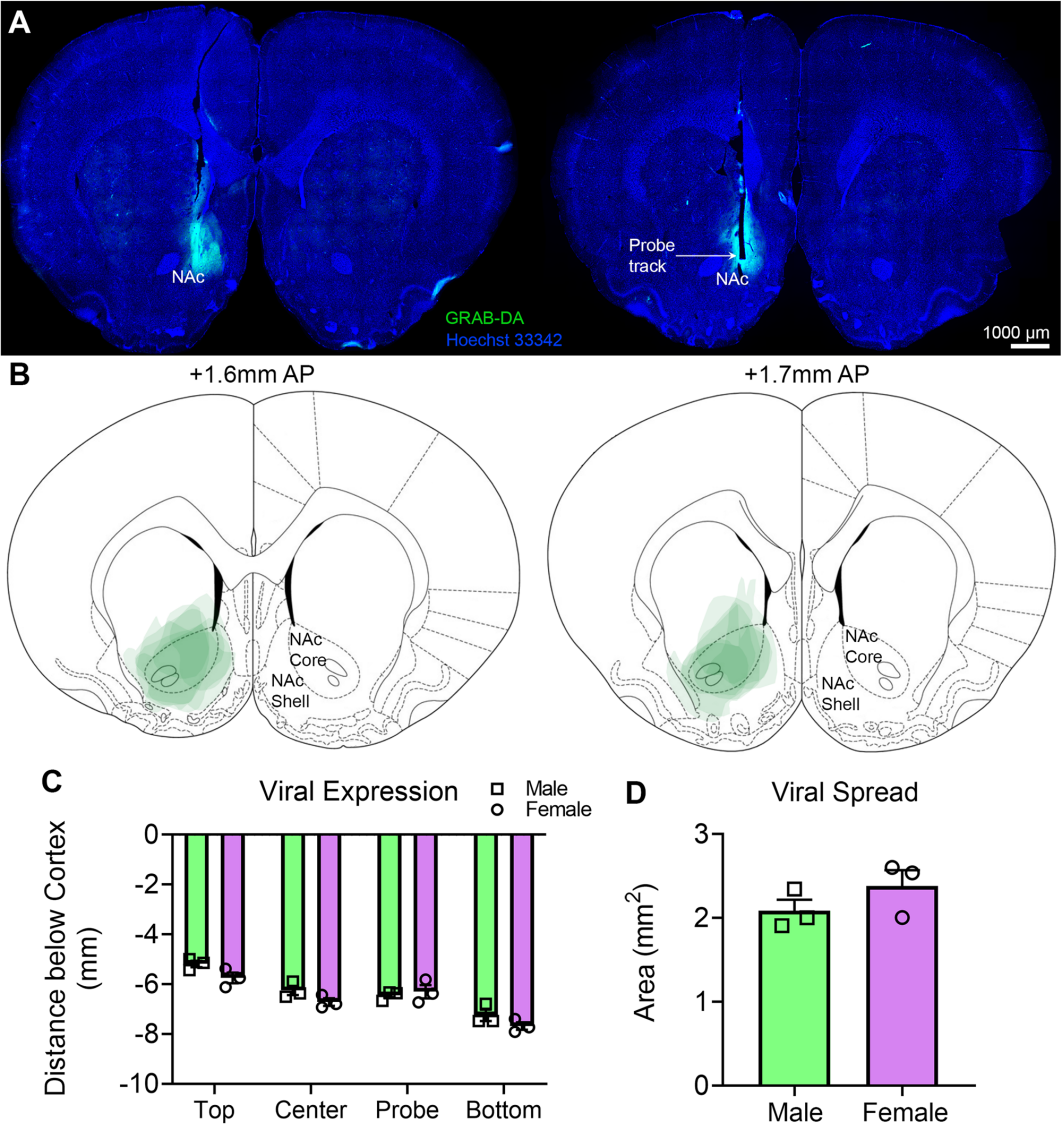
StereoPylot enables accurate targeting of brain regions. ***A***, Representative photomicrographs show GRAB-DA expression and an optical fiber probe track in the NAc of a Long–Evans rat. Hoechst 33342 was used as a counterstain. Scale bar, 1,000 µm. ***B***, Vector cloud maps show that GRAB-DA expression was primarily restricted to the NAc core and shell of each rat. ***C***, Viral expression was quantified by measuring the distance of the cortex to the top, center, and bottom of the viral vector cloud. We also measured the distance between the cortex and the end of the probe track. Our results show that the average distance from the cortex to the vector cloud was as follows: top (−5.47 ± 0.17 mm), center (−6.49 ± 0.15 mm), probe track end (−6.38 ± 0.13 mm), and bottom (−7.47 ± 0.16 mm). ***D***, The average area of the vector cloud (viral spread) did not differ between males (2.08 ± 0.13 mm^2^) and females (2.38 ± 0.18 mm^2^). All graphs depict mean ± SEM. *p < 0.05.

Representative photomicrographs show expression of the fluorescent biosensor GRAB-DA and an optical fiber probe track in the NAc of a male Long–Evans rat that underwent surgery with the StereoPylot instrument (Fig. 12*A*). We also generated viral vector clouds by superimposing the microscope images onto plates from the Rat Brain atlas and drawing the area of viral spread (Fig. 12*B*; Appings et al., 2024). Next, to provide quantitative measures of viral expression, we measured the distance from the top of the cortex to different regions of viral expression (Fig. 12*C*). A two-way ANOVA revealed a main effect of region (*F*_(3,12)_ = 162.6; *p* < 0.0001), no main effect of sex (*F*_(1,4)_ = 1.84; *p* = 0.25), and a significant region by sex interaction (*F*_(3,12)_ = 6.31; *p* = 0.01). However, Sidak's multiple-comparisons test revealed that males and females did not differ in the distance between the cortex and viral vector cloud top (males, −5.19 ± 0.13 mm; females, −5.75 ± 0.21 mm), center (males, −6.26 ± 0.18 mm; females, −6.72 ± 0.15 mm), probe end (males, −6.45 ± 0.11 mm; females, −6.31 ± 0.27 mm), or bottom (males, −7.25 ± 0.23 mm; females, −7.68 ± 0.15 mm; all *p* > 0.18). Lastly, a two-tailed unpaired *t* test revealed no difference in vector spread between male (2.08 ± 0.13 mm^2^) and female (2.38 ± 0.18 mm^2^) rats (*t*_(4)_ = 1.29; *p* = 0.27; Fig. 12*D*). Collectively, these results show that GRAB-DA expression was largely restricted to the NAc core and shell for every rat we analyzed.

## Discussion

Stereotaxic surgery enables precise delivery of drugs, viral vectors, and probes into defined brain regions. As neuroscience experiments increase in complexity (e.g., targeting multiple brain regions within the same animal), the limitations of traditional analog stereotaxic instruments have become more evident. Surgeons must manually read vernier scales for each target, a process that is slow, error-prone, and increasingly impractical. Digital stereotaxic systems address many of these issues but are often prohibitively expensive and are typically offered in rigid configurations with few options for customization or upgrades. Two previous studies have attempted to overcome the limitations of analog stereotaxic instruments by developing robotic stereotaxic devices based on computer numerical controlled (CNC) milling machines (Coffey et al., 2013; Ghanbari et al., 2019). While both of these instruments were effective for stereotaxic surgery, CNC machines are considerably more expensive and less accessible than current consumer-grade 3D printing devices that cost as little as $200 USD. Another study modified an existing motorized stereotaxic frame to perform closed-loop automated craniotomies (Pak et al., 2015), but again, the cost of modifying a fully motorized stereotaxic instrument is considerably higher than modifying an analog stereotaxic instrument as in the current study.

StereoPylot bridges these gaps by using a combination of 3D-printed and commercially available components. StereoPylot is designed to be retrofitted onto a variety of 0.1 mm resolution manipulators and enables fully motorized *X*, *Y*, and *Z* control, adjustable motor speeds (coarse, medium, fine), custom programmable buttons, and a large digital coordinate display housed in a 3D-printed enclosure. Our custom GUI allows surgeons to easily move the manipulator arms automatically by manually entering stereotaxic coordinates or by loading user-defined preset files. StereoPylot delivers repeatable accuracy, reduces repetitive manual operations, and minimizes sources of human error. Because the StereoPylot system is open source, we encourage users to further customize the system to meet evolving experimental needs. In closing, we hope that the neuroscience research community will find StereoPylot to be a feature-rich and affordable alternative to commercial digital stereotaxic instruments.

## Synthesis

Reviewing Editor: Adrien Peyrache, McGill University Decisions are customarily a result of the Reviewing Editor and the peer reviewers coming together and discussing their recommendations until a consensus is reached. When revisions are invited, a fact-based synthesis statement explaining their decision and outlining what is needed to prepare a revision will be listed below. The following reviewer(s) agreed to reveal their identity: Manu Madhav, Gerard Broussard.

Motorized systems for stereotaxic surgeries in animal research improve precision and reproducibility, but the cost of commercially available platforms remains a barrier for many laboratories. This manuscript describes an open-source alternative and provides detailed instructions for constructing and configuring the system at an affordable cost. The approach and its precision were validated both in vivo and in vitro. The manuscript was evaluated by two reviewers, who raised several concerns that must be addressed prior to publication.

Major comments:

1. The instructions regarding the assembly, wiring, and configuration can feel verbose and monotonous. Keeping only high-level instructions in the manuscript and moving the detailed instructions into an assembly guide would improve the readability of the manuscript. This assembly guide can be provided as an appendix to the manuscript and/or on the GitHub page.

2. The in vivo validation is not up to standards for publication. The results state that fluorescence microscopy confirmed GRAB-DA expression in the Nucleus Accumbens core of all 6 rats, yet this was simply mentioned, only supported by a single representative image. Data from all 6 animals should be provided. Furthermore, quantifying and reporting the success rate (e.g. distance from the surface of the brain to the point of highest expression) would be valuable information.

3. Similarly, the in vitro validation seems ad hoc with no quantifiable results. Quantifying the reproducibility of the experiments (e.g. the precision and accuracy of the apparatus after returning to the same position) would also be informative for readers. More generally, the StereoPylot operates as an open-loop system, which lacks the capability to detect or correct for missed steps or mechanical binding. In a surgical context, this introduces an unmanaged risk of targeting error. Rather than relying on the theoretical precision of the NEMA17 motors, empirical data on the system's repeatability should be provided. The authors should perform a back-to-zero hysteresis test. By commanding the unit through a series of complex, multi-axis movements and measuring the deviation upon returning to a calibrated zero point over 50+ trials, the authors can provide a statistically significant measure of final placement repeatability. This should be reported as mean error +/- SEM.

4. The current design requires manually scoring and snapping breadboards and soldering a high-density R-C debouncing circuit with shift registers and Schmitt triggers. This "folded board" approach is prone to short circuits and is difficult for other labs to replicate reliably. Providing Gerber files for a dedicated PCB would significantly enhance the project's accessibility and reliability while avoiding duplicate work for end users. Although this requires some experience with Electronic Design Automation (EDA) software like KiCad or EasyEDA, this should not be too challenging as the circuit was already mapped in Fritzing.

5. The software is pinned to Raspberry Pi OS (Legacy, 64-bit) Bookworm and is explicitly stated to be incompatible with subsequent versions like Trixie. This creates a significant maintenance risk where the instrument cannot be updated to receive critical security patches without breaking the software. This problem can be readily mitigated through the use of containerization or hardware layer abstraction depending on the specific issues that led to pinning the OS.

6. Relatedly, deploying a legacy OS with enabled SSH and Raspberry Pi Connect on an institutional network presents a "Shadow IT" risk. The use of rclone to sync with OneDrive typically involves plain-text token storage. Instructions for obfuscating or encrypting these configuration files should be included and it should be recommended to use dedicated VLAN to further mitigate lateral network infiltration.

7. While an emergency stop button is included, it is software-polled. A physical SPST "Kill Switch" for the 12V motor power must be included for this type of device to prevent catastrophic surgical outcomes in cases where there is a software-side failure during a procedure.

Minor comments:

1. Add manufacturer and part numbers when referring to products (e.g. Easydriver_v44 on line 293)

2. Use full names unless previously abbreviated and defined (e.g. potentiometer instead of pot on line 298)

3. The manuscript specifies Polylactic Acid (PLA) for all 3D-printed components. PLA is known for its low glass transition temperature and susceptibility to warping in lab environments or near heat-generating equipment. The manuscript should include some discussion regarding the mechanical longevity of the motor mounts or provide printing profiles for more durable materials like PETG or ABS.

4. In the Parts List (Table 1), the authors refer to the A4988 driver, but in the text and figure captions, it is variously referred to as A4899 and A899. This should be standardized to A4988.

5. Tapping instructions: The requirement to manually tap M5-0.8 threads into brass couplers is a high-precision task. More detail on how to avoid stripping the 4mm bore would be beneficial for users without mechanical backgrounds.

6. Typographical errors

- Abstract: unquote 'feature-rich'

- Line 72-73: part components -> parts

- Line 159: "Bedian Bookworm" should likely be "Debian Bookworm".

- Line 215: capacitators -> capacitors

- Line 338: "test-harbuttons.py" appears to be a typo for "test-hardbuttons.py".

- Line 339: "test-steppersv3.py" should be checked for consistency with the repository naming conventions.

- Line 340: "VairableList.py" is a typo for "VariableList.py".

- Several scripts mentioned in the text contain typographical errors that could lead to user confusion during the setup process described in Table 4:

* "test-harbuttons.py" should be corrected to "test-hardbuttons.py".

* "VairableList.py" should be corrected to "VariableList.py"

* In the text, Figure 9A is referenced as "Figure 9A- " (missing the end of the range).

## Author Response

Reviewer comments are provided below. We've also highlighted changes to the text documents in yellow to facilitate review of the revised documents.

Synthesis of Reviews:

Computational Neuroscience Model Code Accessibility Comments for Author (Required): N/A Synthesis Statement for Author (Required):

Motorized systems for stereotaxic surgeries in animal research improve precision and reproducibility, but the cost of commercially available platforms remains a barrier for many laboratories. This manuscript describes an open-source alternative and provides detailed instructions for constructing and configuring the system at an affordable cost. The approach and its precision were validated both in vivo and in vitro. The manuscript was evaluated by two reviewers, who raised several concerns that must be addressed prior to publication.

We thank the reviewers for their positive comments and valuable feedback.

Major comments:

1. The instructions regarding the assembly, wiring, and configuration can feel verbose and monotonous. Keeping only high-level instructions in the manuscript and moving the detailed instructions into an assembly guide would improve the readability of the manuscript. This assembly guide can be provided as an appendix to the manuscript and/or on the GitHub page.

We thank the reviewers for this excellent suggestion. We have revised the methods section to place more emphasis on the rationale and major steps of the build process. We have moved the technical and granular portions of the assembly, wiring, and configuration sections to a supplementary Extended Methods document (as suggested by reviewers).

2. The in vivo validation is not up to standards for publication. The results state that fluorescence microscopy confirmed GRAB-DA expression in the Nucleus Accumbens core of all 6 rats, yet this was simply mentioned, only supported by a single representative image. Data from all 6 animals should be provided. Furthermore, quantifying and reporting the success rate (e.g. distance from the surface of the brain to the point of highest expression) would be valuable information.

We agree with the comments raised by the reviewers. The revised manuscript now addresses this shortcoming by quantifying viral expression across all 6 rats. Specifically, we have now added multiple representative photomicrographs from the same animal which show viral expression in the nucleus accumbens (NAc) and an optical fiber probe track which confirms that StereoPylot was able to accurately implant an optical fiber probe into the NAc (Figure 12A). We have also provided vector cloud maps that provide a visual map of viral spread for each animal (Figure 12B), consistent with our previous publications at eNeuro and other journals (references will be added after double-blind peer review). Please note that each vector cloud is semi-transparent, meaning that significant overlap of viral expression across animals in an area such as the NAc will result in a dark green signal, whereas an area with little overlap will be light green. We provided vector clouds for +1.6mm and +1.7mm AP bregma plates, since +1.6mm AP is where GRAB-DA was injected.

To further characterize viral expression, we added distance measurements as suggested by reviewers (Figure 12C). Specifically, we used Zeiss Zen software to acquire whole-tile scans of each brain section and then measured the distance between the top of the cortex and the top, center, and bottom of each vector cloud. We also measured the distance from the top of the cortex to the bottom of the optical fiber probe track for each animal. Our results show that the distance from the cortex to the top of the vector cloud was approximately -5.5mm, whereas the distance to the center of the vector cloud was ~6.5mm, and the distance to the bottom of the vector cloud was ~7.5mm. We also confirmed that the bottom of the optical fiber probe track was approximately -6.4mm below the surface of the cortex. Lastly, we also measured the surface area of the vector cloud for each animal and found that viral spread was comparable between animals (Figure 12D). Taken together, our new results show that GRAB-DA expression and optical fiber probe placements were predominantly within the NAc, validating the accuracy of StereoPylot.

3. Similarly, the in vitro validation seems ad hoc with no quantifiable results. Quantifying the reproducibility of the experiments (e.g. the precision and accuracy of the apparatus after returning to the same position) would also be informative for readers. More generally, the StereoPylot operates as an open-loop system, which lacks the capability to detect or correct for missed steps or mechanical binding. In a surgical context, this introduces an unmanaged risk of targeting error. Rather than relying on the theoretical precision of the NEMA17 motors, empirical data on the system's repeatability should be provided. The authors should perform a back-to-zero hysteresis test. By commanding the unit through a series of complex, multi-axis movements and measuring the deviation upon returning to a calibrated zero point over 50+ trials, the authors can provide a statistically significant measure of final placement repeatability. This should be reported as mean error +/- SEM.

We agree with the Reviewers that empirical data on the accuracy and precision of StereoPylot is warranted for this type of project. We provide new data in Figure 11 which addresses accuracy, precision, and operating temperatures of the stepper motors. The revised Methods section now includes subsections that describe the methods for "Accuracy and precision of StereoPylot" on Line 186 and "StereoPylot operating temperatures" on Line 204. Accuracy was measured by how close the measured value was to the target value, and precision was measured by how close the measures of multiple movements were to each other (calculated as the standard deviation of the measured values).

Initially, we tested these measures by moving each axis 10mm, which represents normal ranges for standard rodent surgery, but we found that there were no missed steps or deviations in our measurements (data not shown). We therefore tested longer working distances (25mm and 30mm) since this would increase the likelihood of missed steps. When measuring precision at 25mm or 30mm target distances, we found that StereoPylot deviated from the target region <0.016mm (16 microns; Figure 11B). Similarly, when we measured accuracy expressed as percent error, we did not observe a single axis measurement exceed 0.08% error, which translates to ~0.02mm (20 microns; Figure 11C). Please note that these ranges exceed the distances transversed during normal rodent surgeries and that users should re-calibrate StereoPylot between surgeries to ensure the highest degree of accuracy and precision.

We provide additional support for accuracy and precision of StereoPylot with video files. In Movie 3, we show that StereoPylot can reliably target regions marked with an ultraprecise manipulator. In Movie 4, we used the pre-set coordinate function to move the manipulator arm to one of our preset hippocampal coordinates. We then lowered the drill and made a craniotomy. We then lifted the drill and assigned StereoPylot to make 25 random movements in each of the 3 axes. After completing random movements, we then re-selected our pre-set hippocampal coordinate and lowered the drill bit to show that it re-entered the craniotomy. We also provide details on the operating temperatures of StereoPylot in Figure 11D. Our results show that the operating temperatures of the stepper motors do not exceed 30 Celsius and therefore display excellent thermal performance. Overall, these new empirical measures validate the accuracy and precision of StereoPylot.

4. The current design requires manually scoring and snapping breadboards and soldering a high-density R-C debouncing circuit with shift registers and Schmitt triggers. This "folded board" approach is prone to short circuits and is difficult for other labs to replicate reliably. Providing Gerber files for a dedicated PCB would significantly enhance the project's accessibility and reliability while avoiding duplicate work for end users. Although this requires some experience with Electronic Design Automation (EDA) software like KiCad or EasyEDA, this should not be too challenging as the circuit was already mapped in Fritzing.

We agree with the Reviewers that a dedicated PCB would improve the accessibility and reliability of the build process. We therefore developed a custom PCB with KiCad V9 software and provided Gerber files to facilitate the build process. A full schematic of the custom PCB is now provided in Figure 6. Notably, the dedicated PCB allowed us to retain all functionality described with our breadboard approach, but the PCB offers additional slots for further expansion and customization of StereoPylot. However, we decided to retain the breadboards for the control box buttons since these connections are not overly complex and adding multiple PCBs to the StereoPylot control box for buttons would significantly increase cost of the build.

5. The software is pinned to Raspberry Pi OS (Legacy, 64-bit) Bookworm and is explicitly stated to be incompatible with subsequent versions like Trixie. This creates a significant maintenance risk where the instrument cannot be updated to receive critical security patches without breaking the software. This problem can be readily mitigated through the use of containerization or hardware layer abstraction depending on the specific issues that led to pinning the OS.

The code for StereoPylot was written while using Bookworm and when we tried to run the software under a fresh Trixie installation, we discovered that the GPIO calls used under Bookworm changed with the Trixie OS to accommodate features of the Raspberry Pi 5. Hardware layer abstraction will not solve the issue as the Raspberry Pi is the interface with the GPIO calls handling the inputs and the outputs. We have found in the OpenSource community that sometimes core changes are implemented that can fundamentally break functionality. We have experienced this scenario in other Python based projects trying to maintain CUDA processing with DeepLabCut and in using PiCamera dependent modules on the newest Python releases. In these situations, we find that the most effective solution is to accurately define the software installation steps that allow the unit to function as intended, rather than write entirely new code. To address this issue, we provide requirements.txt and localrequirements.txt files in our Extended Data to ensure software version compatibility.

6. Relatedly, deploying a legacy OS with enabled SSH and Raspberry Pi Connect on an institutional network presents a "Shadow IT" risk. The use of rclone to sync with OneDrive typically involves plain-text token storage. Instructions for obfuscating or encrypting these configuration files should be included and it should be recommended to use dedicated VLAN to further mitigate lateral network infiltration.

We agree with the concerns about deploying a legacy OS on institutional networks and the caveats of using rclone to sync with OneDrive. While the initial setup of the Raspberry Pi requires a WiFi connection, most institutions offer guest or public WiFi which limit security risks. Alternatively, a mobile phone hotspot can be used to assist with the initial setup.

Aside from the initial setup needing to pull the install packages from the python repository and StereoPylot files from GitHub, StereoPylot does not require an internet connection to function. This is a point we did not make fully transparent in our original submission. We have added text to the Extended Methods that recommends users consult with their IT department about security concerns, since policies differ by institution. Moreover, the rclone commands are not part of the core StereoPylot code and reside in the startup script and do not need to be utilized. To address the rclone issue, we have added a USB adapter cable to the StereoPylot control box so that users can manually transfer coordinate lists offline. We feel that this is a simple solution that allows users to operate StereoPylot completely offline, minimizing "shadow" IT risks.

7. While an emergency stop button is included, it is software-polled. A physical SPST "Kill Switch" for the 12V motor power must be included for this type of device to prevent catastrophic surgical outcomes in cases where there is a software-side failure during a procedure.

The reviewers raise a fair point. The Emergency button is hardwired to the Raspberry Pi and uses event detection to override all commands to terminate motor movement. This function allows for recovery of function while retaining relative zero settings which can be useful but, admittedly, it is not enough. Therefore, we have included a prominently positioned SPST switch to cut all power to StereoPylot and halt movement instantly (see Figure 1A-B).

Minor comments:

1. Add manufacturer and part numbers when referring to products (e.g. Easydriver_v44 on line 293) We have updated the main text to include part numbers and manufacturers whenever possible. Table 1 is also referenced at the start of the 'Methods' section which provides a comprehensive list of all items that are required with their corresponding supplier and catalogue/product number.

2. Use full names unless previously abbreviated and defined (e.g. potentiometer instead of pot on line 298) We have updated the manuscript accordingly.

3. The manuscript specifies Polylactic Acid (PLA) for all 3D-printed components. PLA is known for its low glass transition temperature and susceptibility to warping in lab environments or near heat-generating equipment. The manuscript should include some discussion regarding the mechanical longevity of the motor mounts or provide printing profiles for more durable materials like PETG or ABS.

We agree that despite its widespread use, PLA can warp, become brittle over time, and should be used cautiously with equipment that generates heat. Although we have not experienced any of these issues with >6 months of StereoPylot use, we acknowledge that other filaments with superior strength and thermal resistance would be ideal. The one caveat is that not all commercial printers are capable of printing alternative filaments. Nonetheless, we have now provided additional text in the revised manuscript (Lines 104-108) which recommend using stronger materials such as PETG or ABS. We also confirmed that our motor mount files accurately print with PETG and ABS filament (Figure 3D-E). We also note that users should regularly check the 3D motor mounts and replace them if there are any signs of mechanical wear (e.g., cracks). Importantly, our new operating temperature data (Figure 11D), show that the operating temperature under load is still well-below the glass transition temperature of PLA (~55-65 {degree sign}C).

4. In the Parts List (Table 1), the authors refer to the A4988 driver, but in the text and figure captions, it is variously referred to as A4899 and A899. This should be standardized to A4988.

We apologize for this oversight. The text has been updated accordingly.

5. Tapping instructions: The requirement to manually tap M5-0.8 threads into brass couplers is a high-precision task. More detail on how to avoid stripping the 4mm bore would be beneficial for users without mechanical backgrounds.

We agree that thread tapping may be challenging for individuals without mechanical backgrounds. Our revised submission now provides a supplementary video file (Movie 2) that provides instructions on how to successfully tap a thread. We have also added a sentence to the legend in Figure 9 that users should avoid tapping at an angle or overtightening to avoid stripping the bore.

6. Typographical errors - Abstract: unquote 'feature-rich'

- Line 72-73: part components -> parts - Line 159: "Bedian Bookworm" should likely be "Debian Bookworm".

- Line 215: capacitators -> capacitors - Line 338: "test-harbuttons.py" appears to be a typo for "test-hardbuttons.py".

- Line 339: "test-steppersv3.py" should be checked for consistency with the repository naming conventions.

- Line 340: "VairableList.py" is a typo for "VariableList.py".

- Several scripts mentioned in the text contain typographical errors that could lead to user confusion during the setup process described in Table 4: * "test-harbuttons.py" should be corrected to "test-hardbuttons.py". * "VairableList.py" should be corrected to "VariableList.py" * In the text, Figure 9A is referenced as "Figure 9A- " (missing the end of the range).

These errors have been corrected.
