# Supplementary material for: StereoPylot: An Open-Source Raspberry Pi-Based Stereotaxic Apparatus Controller with 3D Printed Components for Fully Motorized Control, Digital Display, and Customizable Features
Source: eNeuro. 2026 Jul 7;13(7):ENEURO.0460-25.2026. doi: 10.1523/ENEURO.0460-25.2026 (PMC13362190; doi:10.1523/ENEURO.0460-25.2026)
Supplement: Data 1 — All relevant code and files for StereoPylot. Download Data 1, ZIP file. [file eneuro-13-ENEURO.0460-25.2026-s007.zip › StereoPylot-main/Build_Setup_Files/Button_Overlay/Button_Overlay.pdf]

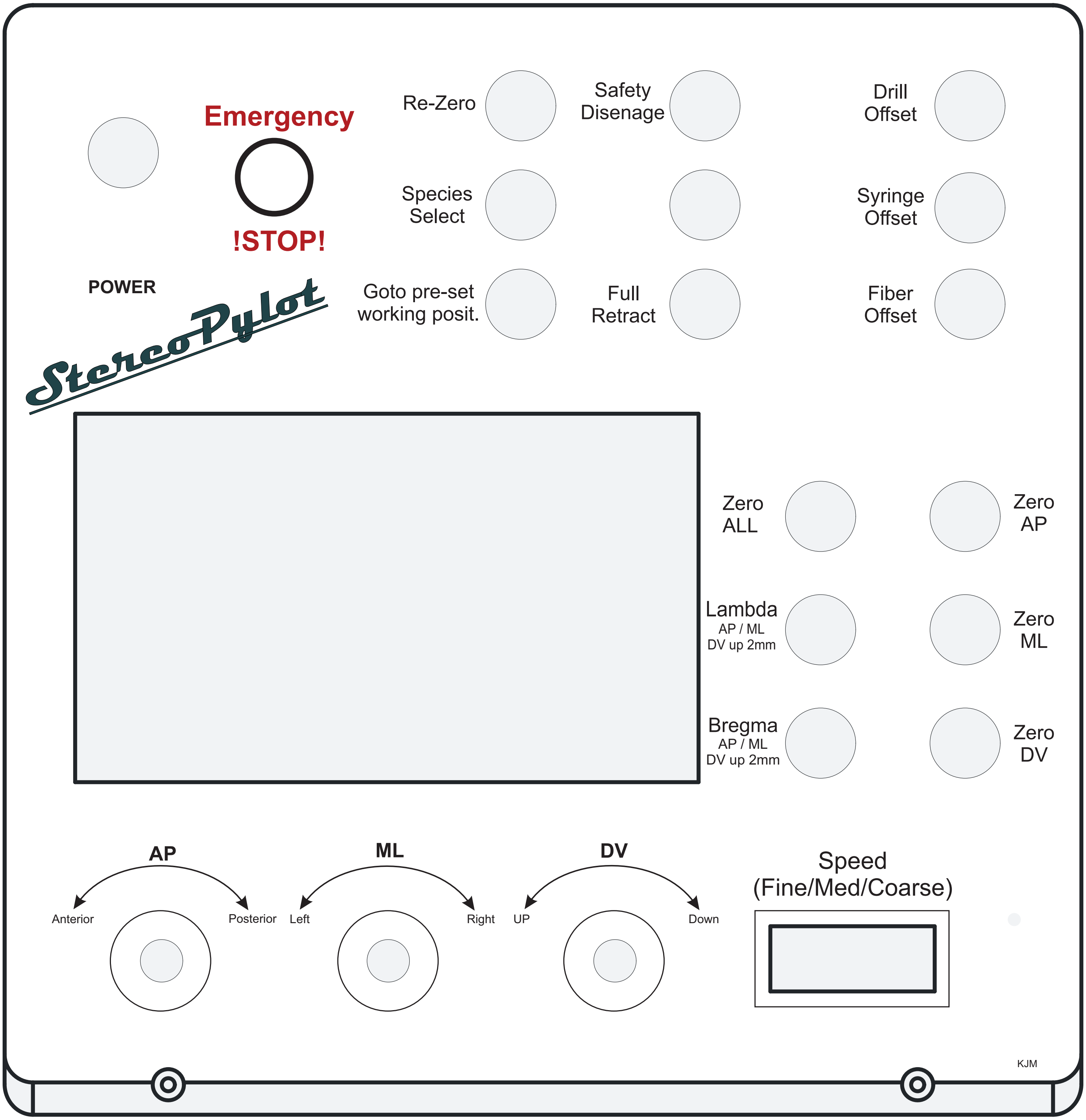
Emergency
!STOP!
POWER
StereoPylot
Re-Zero
Safety Disenage
Drill Offset
Species Select
Syringe Offset
Goto pre-set working posit.
Full Retract
Fiber Offset
Zero ALL
Zero AP
Lambda AP / ML DV up 2mm
Zero ML
Bregma AP / ML DV up 2mm
Zero DV
AP
Anterior
Posterior
ML
Left
Right
DV
UP
Down
Speed (Fine/Med/Coarse)
KJM
