# Supplementary figures and images for: StereoPylot: An Open-Source Raspberry Pi-Based Stereotaxic Apparatus Controller with 3D Printed Components for Fully Motorized Control, Digital Display, and Customizable Features
Source: eNeuro. 2026 Jul 7;13(7):ENEURO.0460-25.2026. doi: 10.1523/ENEURO.0460-25.2026 (PMC13362190; doi:10.1523/ENEURO.0460-25.2026)

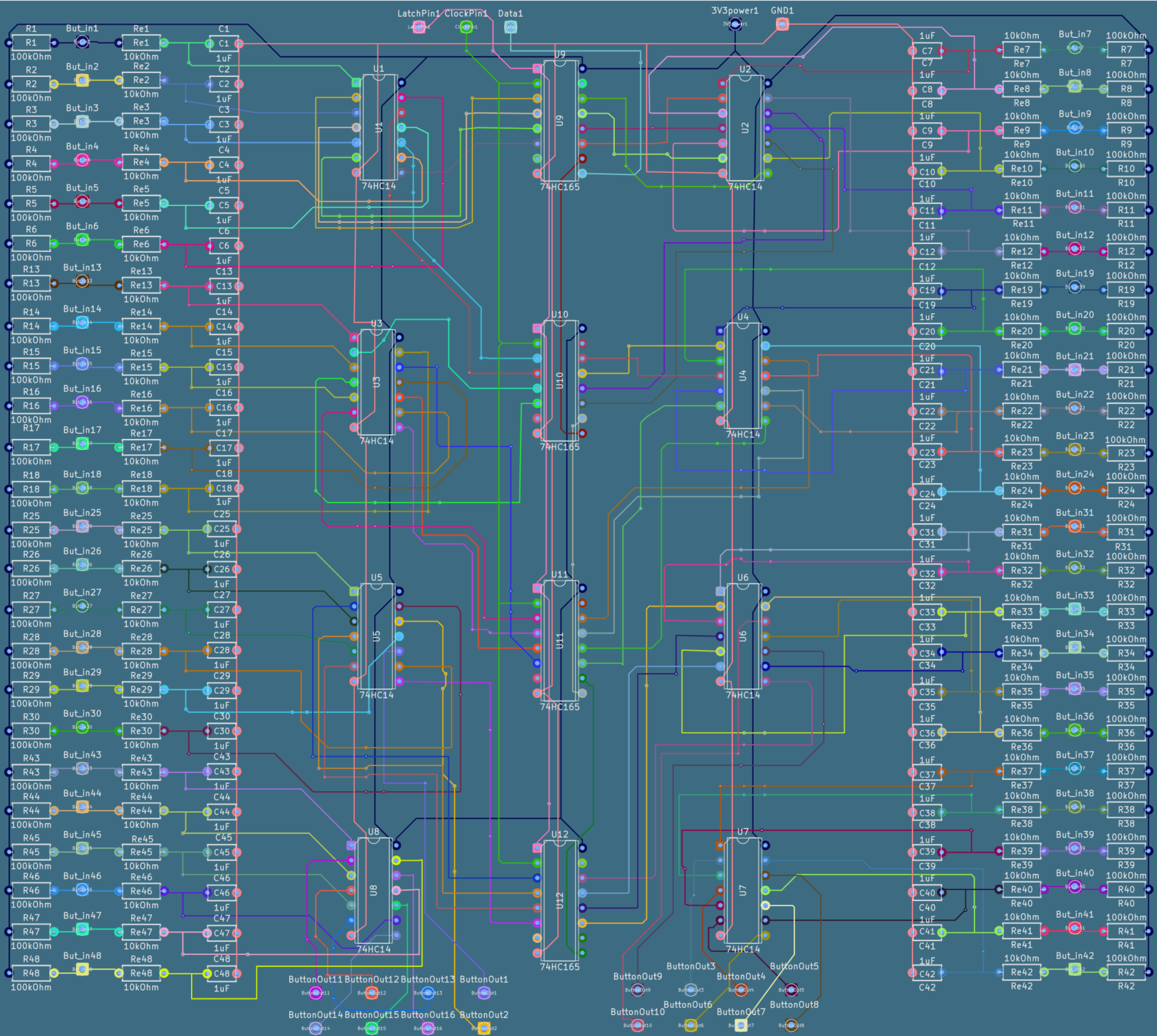

Supplement: Data 1 — All relevant code and files for StereoPylot. Download Data 1, ZIP file. [file eneuro-13-ENEURO.0460-25.2026-s007.zip › StereoPylot-main/Build_Setup_Files/Wiring/PCB board schematic.pdf]

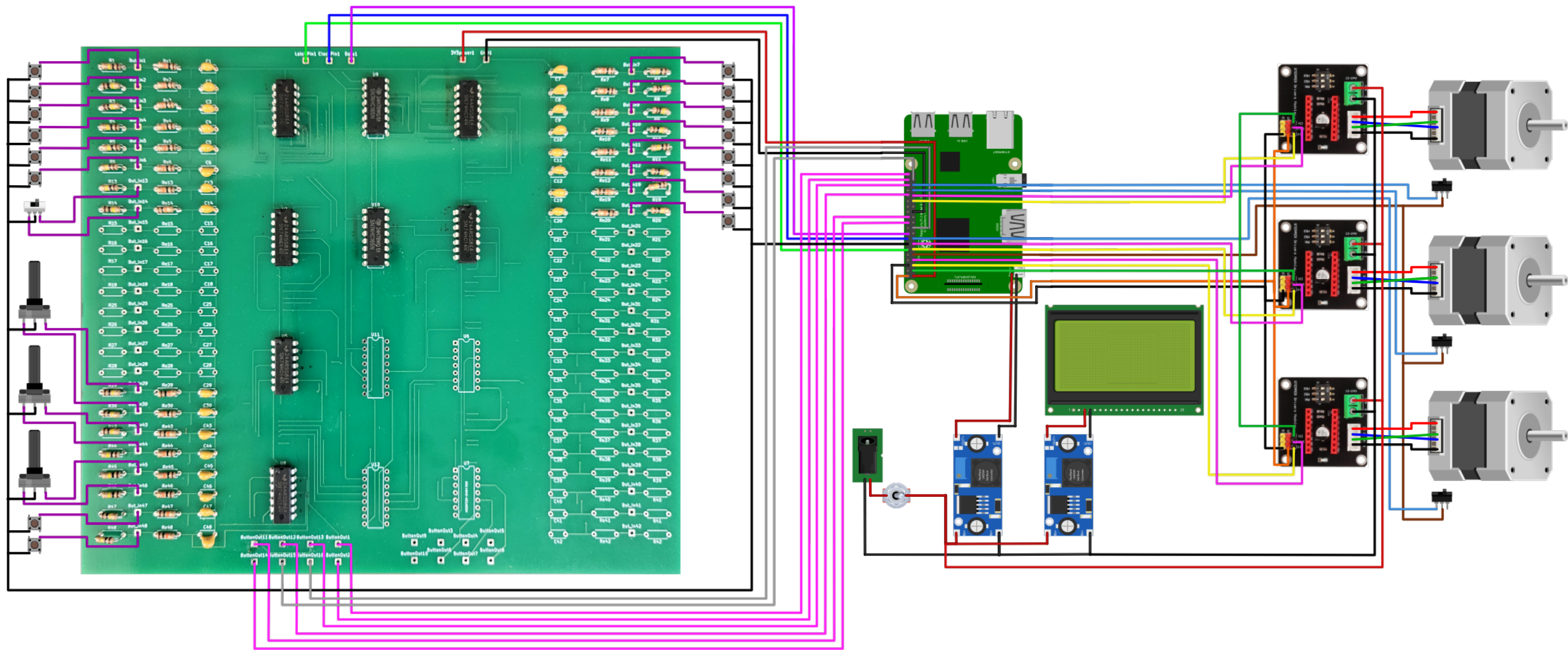

Supplement: Data 1 — All relevant code and files for StereoPylot. Download Data 1, ZIP file. [file eneuro-13-ENEURO.0460-25.2026-s007.zip › StereoPylot-main/Build_Setup_Files/Wiring/Wiring Diagram.pdf]

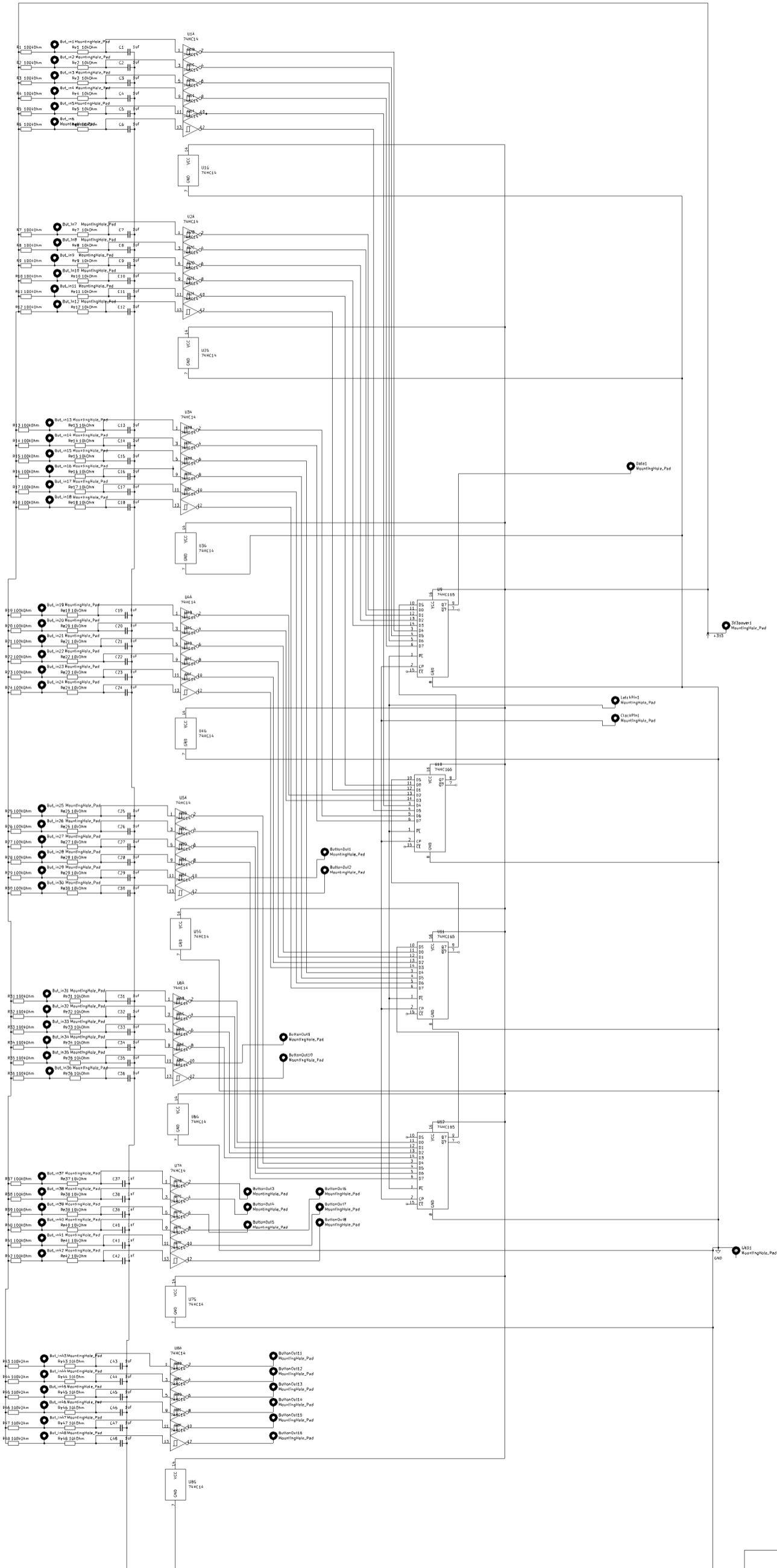

Supplement: Data 1 — All relevant code and files for StereoPylot. Download Data 1, ZIP file. [file eneuro-13-ENEURO.0460-25.2026-s007.zip › StereoPylot-main/Build_Setup_Files/Wiring/Wiring Schematic.pdf]
