# Supplementary material for: StereoPylot: An Open-Source Raspberry Pi-Based Stereotaxic Apparatus Controller with 3D Printed Components for Fully Motorized Control, Digital Display, and Customizable Features
Source: eNeuro. 2026 Jul 7;13(7):ENEURO.0460-25.2026. doi: 10.1523/ENEURO.0460-25.2026 (PMC13362190; doi:10.1523/ENEURO.0460-25.2026)
Supplement: Data 2 — This document provides additional technical details for the StereoPylot build process. Download Data 2, DOCX file. [file eneuro-13-ENEURO.0460-25.2026-s002.docx]

**Raspberry Pi Setup**

The initial setup of the Raspberry Pi should be done prior to mounting the unit in the control box. Download Raspberry Pi Imager software (<https://www.raspberrypi.com/software/>) with a personal computer and transfer the files to a 64GB microSD card using a USB to microSD adapter. The image writing process consists of the following steps:

- Within the Raspberry Pi Imager software under ‘Raspberry Pi Device’ select: ‘Raspberry Pi 3’
- Under ‘Software’ select: ‘Raspberry Pi OS (other)’
- Select: ‘Raspberry Pi OS (Legacy, 64-bit) – A port of Debian Bookworm…’
- Select the location of the microSD card under Storage and click: ‘next’
- Select: ‘Apply OS Customization Settings’ and complete the Hostname, User and Pass fields.
- Save and write to the microSD.

These instructions are current at the time of writing, but the imager software may receive updates over time. It is important that the 64bit version of Bookworm be installed and not Trixie or later versions as there are fundamental changes in how Trixie manages the input and output pins which are currently not compatible with the StereoPylot software. Remember to take note of your Hostname, User, and Pass fields for future reference.

During the initial setup, the Raspberry Pi should be connected to a standard display monitor via a HDMI cable the imaged microSD card is inserted into the microSD slot. A wireless keyboard and mouse combo was connected to the Raspberry Pi via a USB dongle and a 5V 3A power source was used to power the Raspberry Pi. After the initialization process completed and the user desktop environment was loaded, Wi-Fi was set up to finalize the installation process. The Wi-Fi settings are located in the upper right corner of the desktop. Before connection to an institutional network contact your local IT department to discuss any concerns they may have with open source projects on the network. Notably, most institutions provide guest Wi-Fi access that should be sufficient for the installation process while minimizing IT risks. The final installation size is approximately 300MB, so a mobile hotspot is another feasible option for a temporary internet connection if institutional Wi-Fi options are limited.

Using the command line terminal, enter the following commands below to update the Raspberry Pi and complete set up of the virtual environment:

- sudo apt update
- sudo apt full-upgrade
- python -m venv --system-site-packages venv1
- *Optional Step:* Install Raspberry Pi Connect as per official documentation found on the Raspberry Pi website. (<https://www.raspberrypi.com/documentation/services/connect.html>).

Once the step above is complete, the Raspberry Pi can be powered down and the microSD card can be transferred to a personal computer. Use the file management and text editing software to find and open the following file:

- /boot/firmware/cmdline.txt

Within the cmdline.txt file there is a line of text which initializes the Raspberry Pi when booted. To configure the correct resolution for most 5” LCD screen enter the following at the front of the line of text:

- video=HDMI-A-1:800x480

The microSD card can then be transferred back to the Rasperry Pi and powered on to set the resolution for the 5” LCD display.

- Under: ‘Screen Configuration’, select ‘Preferences’ and change the resolution to 800x480.

The Raspberry Pi should then be powered off and set aside until needed.

**Assembly of the StereoPylot control box**

Print the button overlay with vinyl sticker paper and use a blade to cut the holes for the LCD display and buttons. Once the vinyl button overlay was attached to the face of the upper control box, attach the 3-way switch (Amazon, #B085ZPD1FM) and power switch (Amazon, #B0CGTJXV1Y) as shown in **Figure 4A**. The 5” LCD screen can then be mounted onto the backside of the upper control box and secured with washers and 4mm long M2 bolts (Figure 4B). A flat cable HDMI connector (Amazon, #B07R6CWPH1) can then be plugged into the HDMI port on the top of the LCD display (**Figure 4B**).

Next, the 5cm x 7cm PCB breadboards should be cut to the approximate sizes listed in **Table 1** by scoring the board with a razor blade and snapping the breadboard over a metal ruler. The control box buttons (Amazon, #B01E38OS7K) and rotary encoders (DigiKey, EC11E18244AU) are then arranged and soldered to the breadboards as shown in **Figure 4C**. Fitment of the buttons to the control box face was tested and, if required, the holes of the control box can be enlarged slightly by inserting the blade of a pair of scissors and rotating gently. Three pins are present on the encoders, the ground pin is the middle pin and the outer pins are the data pins. For the buttons, pins on opposite corners make a circuit when the button is pressed. To make a ground connection, the buttons and encoders are connected in series with 24ga wire and a ~20cm length of 24ga wire was soldered to the last connection in the series. The other end of the 20cm wire was soldered to the T-type GPIO expansion board (Amazon, #B089SXW3HD) pin labelled ‘Button Board GND’ in the provided Raspberry Pi pin layout (**Table 2)**. A 35cm length of 24ga wire was then soldered to the opposite corner of each button and to the data pins of each encoder (**Figure 4D**). Please note that these wires will need to be attached to the printed circuit board (PCB) later in the build process. The button breadboards were then installed on the upper control box with washers and 4mm long M2 bolts. A drill bit was used to drill small holes in breadboards so that they would align with the holes in the back of the upper control box (**Figure 4E**).

**PCB Assembly**

A custom PCB was designed using KiCad V9 software (<https://www.kicad.org/>) and prototypes were ordered from PCBasic ( <https://www.pcbasic.com/>). After uploading the Gerber files we made the following selections:

- Material type: FR-4
- Layer count: 2
- TG: TG150
- Size: 178.00 x 204.00mm
- Board type: single piece
- Thickness: 1.6mm
- Outer copper weight: 1oz
- Inner copper weight: 0.5oz
- Minimum trace width/spacing: 10mil
- Minimum drill hole: 0.3mm
- Test method: Flying probe testing
- Surface finish: HASL lead free
- Profiling Method: Mechanic molding
- Pre-plating process: Electroless copper plating

The PCB board circuits were checked with a multimeter and the components were soldered in place (**Figures 5, 6, 10**). Two SN74HC165N shift registers (DigiKey, #296-8251-5-ND) were installed in the U9 and U10 positions. Positions U1, U2, U3, U4, U5 and U8 were filled with SN74HC14 Schmitt triggers (DigiKey, #296-1577-5-ND). 100kOhm resistors (DigiKey, #CF14JT100KCT-ND) were soldered in positions R1 to R14, R19, R20, R29, R30, and R43 to R48. 10kOhm resistors (DigiKey, #CF14JT10K0CT-ND) were installed in positions Re1 to Re14, Re19, Re20, Re29, Re30, and Re43 to Re48. Finally, 1uF capacitors (DigiKey, #399-9886-1-ND) were soldered to positions C1 to C14, C19, C20, C29, C30, and C43 to C48. Five ~35cm lengths of 22ga wire were soldered to the PCB at the 3v3Power1, GND1, LatchPin1, ClockPin1 and DataPin1 solder pad holes. Eight ~35cm lengths of 24ga wire were soldered to the pads labelled ‘ButtonOut1’, ‘ButtonOut2’ and ‘ButtonOut11’ to ‘ButtonOut16’

**Power supply wiring harness**

A short length of 18ga wire was soldered to the load side of the power switch and five ~20cm lengths of 20ga wire were attached to the other end of the wire. These five lengths of wire were connected to the VIN connection of the three motor driver expansion boards (Amazon, #B08RP2SCJ7) and to the IN+ connection of the two LM2596S DC-DC stepdown buck converters (Amazon, #B07PCSL919). Five additional ~30cm lengths of wire were connected to the GND connection of the motor driver expansion boards and to the IN- side of the LM2596S DC-DC stepdown buck converters. These wires are later connected to the black lead from the power jack (**Figure7**).

Both LM2596S DC-DC stepdown buck converters were adjusted to 5.1V output using a multimeter connected to the Out+ and Out- pads and turning the brass screw on the adjustable resistor. One buck converter is used to power the Raspberry Pi. A 15cm length of 22ga wire was used to connect the Out+ of the buck converter to the PP2 test pad on the Raspberry Pi 3b+ (yellow wire in **Figure 7B)**, whereas a second wire was used to connect the Out- of the buck converter to test pad PP5 of the Raspberry Pi. (green wire in **Figure 7B)**. Two ~15cm 24ga wires were then soldered to the positive and negative pads of a micro-USB plug (Amazon, #B0BR8V2X1B) (**Figure 7C**). These leads were then connected to the second buck converter and the micro-USB jack was used to power the LCD screen (**Figure 7D**).

Next, the 3D printed lower control box was attached to the middle control box with 6mm M3 screws and the 5.2mmx2.5mm female power jack (Amazon #B0F28MB721) was installed (**Figures 7A-C**). The red (positive) lead was extended by attaching a length of 18ga wire and then soldering it to the supply side of the power switch. The Raspberry Pi was then mounted to the lower control box with 4mm M2 screws and the ground wire for the buck converters, and motor driver expansion boards were then attached to the black lead of the power jack (**Figure 7D**). We then cut a hole in the vent side of the control box to accommodate a USB adapter for offline file transfers (Amazon, #B00S2N2Q4U). This hole was then covered with a 3D printed cable holder and secured with hot glue (**Figures 7E-F**).

**Modifying Stereotaxic Manipulators**

A brass shaft coupler (Amazon, #B0D3TKG5WN) was used to connect the drive steppers to the medial-lateral and dorsal-ventral axis manipulators. An Allen key was used to loosen the set screw on the side of the manipulator adjustment knobs which then could be then turned off the drive shaft. The 4mm bore of the coupler was then tapped to screw onto the drive shaft. The set screws were then removed from the coupler and the 4mm bore side was inserted into the 3D printed tapping guide (**Figure 9A**). A benchtop vice was used to hold the guide and coupler firmly in place while a M5-0.8 thread tap was used to create threads; this process was lubricated with a drop of light machine oil (**Movie 2**). The 20-tooth gear with the 4mm bore (Amazon, #B078Z7ZGGF) was tapped in a similar fashion but without the use of a tapping guide. Once tapped, the brass coupler was screwed onto the drive shaft and secured with set screws (**Figure 9B**).

The left and right motor mounts were test fit and, if necessary, a thin strip of electrical tape was wrapped around the oblong base of the manipulator to create a snug fit (red arrow in **Figure 9C**). With one half of the mount in place, the stepper motor (Amazon, #B0D1QFFHDK) was inserted and the coupler set screws were tightened**.** The other half of the motor mount was then put in place and four 25mm M2 bolts and nuts were used to secure the motor mount (red arrows in **Figure 9D**). Two 12mm M3 bolts were used to secure the stepper motor in opposing corners (black arrows in **Figure 9D**). Three limit switches (Amazon, #B0C8THL9NV) were wired in the normally open (NO) configuration using 22ga wires that were approximately 20cm longer than the wires that came with the stepper motors. The wires were threaded through the channel in the motor mount until the switch was seated in the recess; a small dab of hot glue was used to prevent the switch from moving (**Figure 9E**). Installing the rear motor mount consisted of removing the adjustment knob of the anterior-posterior manipulator and then the rectangular faceplate (**Figure 9F**). The 3D printed motor mount was then slid over the end of the manipulator, and the face plate was re-attached (**Figure 9G**). The threaded 20-tooth gear was then screwed onto the drive shaft and its set screw was tightened (red arrow in **Figure 9G**). Four 8mm M3 bolts were used to secure the stepper motor to the rear mount and a 5mm bore 20-tooth gear (Amazon, #B078Z6YZCY) was affixed to the shaft. The gears were connected using a 6mm wide 142mm GT2 belt (Amazon, #B0BZNNV1J9). Braided sleeving (Amazon, #B07S81TNXL) was used to cover the wires from each stepper.

**Finalizing the wiring**

An A4988 stepper driver module (Amazon, #B08RP2SCJ7) was inserted into each motor expansion board ensuring that the VMOT labelled pin of the driver aligns with the VIN pin of the expansion board. Current output of the driver was adjusted by turning the resistor potentiometer to completely clockwise and then backing off approximately one quarter of a turn ( **Figure 10A & 10B**). Then, the motor driver expansion boards were connected to a T-type GPIO expansion board with 24ga wire (**Figure 10C**). Five 20cm wires were soldered to the V, G, DIR, EN, and STEP pins of each motor expansion board. The free ends of all three ‘EN’ wires were connected to a 5cm length of 22ga wire which was then soldered to the GPIO expansion board in the ‘Enable ALL’ position (**Table 2**). The process was repeated with the V (5V) and G (GND) wires which were connected to the ‘stepper 5V’ and the ‘stepper GND’ positions respectively. The DIR and STEP wires for each motor expansion board were soldered to their corresponding position on the GPIO expansion board indicated by the Raspberry Pi pin layout (**Table 2**). The drivers were set to half stepping by switching MS1 to the ON position and leaving MS2 and MS3 in the OFF position (**Table 3**). The stepper motors and the limit switch wires were then threaded through the side of the control box. One wire from each pair of limit switch wires was soldered to a 5cm length of 22ga wire to create a common ground which was soldered to the ‘Limit GND’ pin of the GPIO expansion board. The remaining limit switch wires were soldered to the corresponding pins. The stepper wires were then plugged into the motor expansion boards.

Wires from the buttons were then threaded through the bottom of the control box and soldered to the pads labelled ‘But_in1’ to ‘But_in14’, ‘But_in19’ and ‘But_in20’. The ‘emergency stop’ and the ‘safety’ buttons were connected to ‘But_in29’ and ‘But_in30’ and the encoder wires were soldered to pads ‘But_in43’ to ‘But_in48’ (**Figure 10D**). The wires from the PCB board were then passed through the bottom of the control box and soldered to the GPIO expansion board. ‘ButtonOut1’ and ‘ButtonOut2’ connect to ‘EMERG STOP’ and ‘Safety button’ on the GPIO expansion board and ‘ButtonOut11’ to ‘ButtonOut16’ connect to the ‘Encoder’ pins. The 3v3Power, GND, Latch, Clock, and Data were also connected to the GPIO expansion board according to the map in **Table 2**. The wires were bundled in groups (**Figure 10D**) and then gently pulled back into the control box so the PCB board could lay flat (**Figure 10E**). Four 4mm M3 screws were used to secure the base cover (**Figure 10F**).
